# Cultural adaptation and evaluation of the measurement properties of the Facilitator Competency Rubric for clinical simulation facilitators

**DOI:** 10.1590/1518-8345.7214.4257

**Published:** 2024-07-29

**Authors:** Daniel Gonçalves Campos, Juliany Lino Gomes Silva, Ruana Luiz Ferreira da Silva, Angélica Olivetto Almeida, Ana Luísa Brandão de Carvalho Lira, Ana Railka de Souza Oliveira-Kumakura

**Affiliations:** 1 Universidade Estadual de Campinas, Faculdade de Enfermagem, Campinas, SP, Brazil.; 2 Universidade Federal do Ceará, Departamento de Enfermagem, Fortaleza, CE, Brazil.; 3 Université Paris Cité, Département Universitaire en Sciences Infirmières, Paris, France.

**Keywords:** Simulation Training, Surveys and Questionnaires, Education, Teaching, Faculty Professional Competence

## Abstract

**Objective:**

translate and adapt the Facilitator Competency Rubric to the Portuguese language and the Brazilian culture, and analyze the measurement properties.

**Method:**

methodological study that completed the steps of translation, synthesis of translations, back translation, review by a Committee of Experts composed of 7 professionals, testing of the pre-final version with 33 simulation facilitators, and submission to the author of the original instrument. For content validation, the Content Validity Index and the modified Kappa Coefficient were calculated. For reliability, Cronbach’s α and the Intraclass Correlation Coefficient were evaluated by 52 and 15 simulation facilitators, respectively.

**Results:**

two rounds of content evaluation were carried out, resulting in changes to 19 items in the first evaluation and 3 items in the second. The overall scale achieved a Cronbach’s α of 0.98 and an Intraclass Correlation Coefficient of 0.95 to 0.97.

**Conclusion:**

the Facilitator Competency Rubric was translated and culturally adapted to the Brazilian reality and presented content validity, reliability and stability, with safe results for use in teaching and research.

## Highlights

**(1)** The Brazilian version of the FCR has valid content and is reliable for national use.

**(2)** The FCR adapted to the Brazilian context measures the competence of the simulation facilitator.

**(3)** Measuring competence with the FCR favors the scenario of teacher education in simulation.

## Introduction

 Simulation-based learning has had an advent in recent years and has been a teaching strategy widely used by undergraduate programs in different areas of health ^(^ ^1^ ^-^ ^2^ ^)^ . Researchers point out that approximately half of the hours referring to traditional clinical experience can be replaced by simulated clinical experiences if certain conditions for good execution are met, such as formal training in simulation pedagogy, an adequate number of teachers to assist students, the presence of specialists in conducting a debriefing based on evidence and appropriate material resources to make the environment as realistic as possible ^(^ ^2^ ^)^ . 

 To this end, different measurement instruments have been developed over the years to evaluate elements that are necessary for the implementation of this teaching strategy or to evaluate its results, especially those of participants ^(^ ^3^ ^)^ . For example, the National League for Nursing (NLN) and the International Nursing Association for Clinical and Simulation Learning (INACSL) provide instruments that have been translated and validated for different cultures and that focus on evaluating skill performance, satisfaction, perception of the educational experience, knowledge/learning, critical thinking/clinical judgment, self-confidence/self-efficacy, debriefing, facilitator competence, and on assessing the level of organization regarding the design of the simulated scenario ^(^ ^4^ ^)^ . 

 Most of these instruments evaluate the student’s experience with the simulation, however, the evaluation of the individual who facilitates the simulation is still undefined, despite evidence of their performance being included in other types of tools ^(^ ^5^ ^)^ . Among the scales focusing on the facilitator is the Facilitator Competency Rubric (FCR). It was developed to assess and measure the educator’s level of competence in facilitating learning using simulation, and presents versions in English and German. Benner’s theoretical framework is used to differentiate the facilitator’s competency levels, represented by: novice, advanced beginner, competent, proficient and expert ^(^ ^5^ ^)^ . 

 The facilitator is considered the professional educator who is responsible for monitoring and ensuring the entire simulation-based experience in the safest way possible so that learners work cohesively, focusing on understanding the learning objectives and developing a plan to achieve the desired results. Being a facilitator requires training and skills in leading, supporting and finding ways to help learners achieve expected results ^(^ ^6^ ^)^ . 

 The beginner facilitator is an inexperienced person whose behaviors and decisions are guided by pre-established rules and who can learn the tasks, but does not know what to do with the information obtained. The advanced beginner presents an acceptable performance. The competent is able to develop, implement and prioritize an action strategy to resolve a problem, feeling in control of the situation. The proficient facilitator is able to see the situation from a more global perspective based on their experience, acting more quickly and flexibly compared to the competent one. Finally, the expert is not based on rules, but on their experience and intuition. Within the education and teaching setting, professionals may move through these levels as they change job responsibilities or learn new teaching strategies ^(^ ^5^ ^)^ . 

 The FCR instrument is composed of five dimensions: preparation, prebriefing, facilitation, debriefing and evaluation. For these dimensions, there are a total of 29 items, which can be classified on an ordinal scale from beginner (1) to advanced beginners (2); competent (3); proficient (4) to expert (5). The scale development study found that inter-rater reliability was good. Generalization coefficients (G), used to evaluate inter-rater reliability and to determine the amount of variance attributable to them, ranged from good to excellent (0.80 to 0.99), and the items deadline, day of the week, time of the day and types of simulation were significant predictors of the instrument’s global score ^(^ ^5^ ^)^ . 

 From the perspective that the FCR is an instrument that, in the Brazilian context, can provide guidance for the training of teaching staff and be used as a self-assessment tool or formative or summative evaluation of a simulation facilitator ^(^ ^5^ ^)^ , it is proposed to carrying out this study. The objective was to translate and adapt the Facilitator Competency Rubric to the Portuguese language and Brazilian culture, and to analyze the measurement properties. 

## Method

### Study design

**Figure 1:**
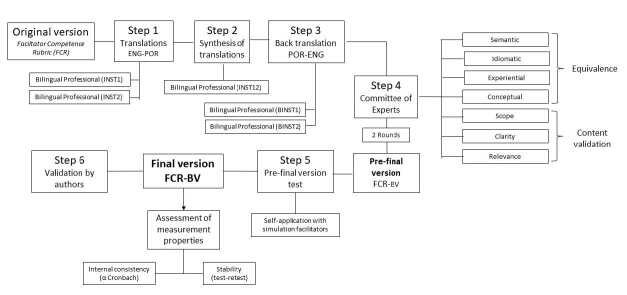
- Methodological flowchart of the study. Campinas, SP, Brazil, 2023

 Methodological study, which completed the following steps: translation, synthesis of translations, back translation, review by a committee of experts, testing of the final version and submission to the author of the original instrument ^(^ ^7^ ^)^ . In addition, content validation and reliability measurement were carried out. The research was described based on the criteria adopted by the COnsensus-based Standards for the selection of health Measurement INstruments (COSMIN) checklist ^(^ ^8^ ^)^ . Figure 1 elucidates the methodological flowchart of the study. 

## Period

The data collection period occurred after the study was approved by the Research Ethics Committee and was carried out between April and September 2023.

### Step I – Translations

 The instrument was translated from English to Brazilian Portuguese, obtaining two initial versions: one made by a translator with knowledge of simulation and health terminologies and the other made by a translator with no knowledge in the area. Both professionals are fluent in the original language of the instrument and are originally from Brazil, with experience in the target language of the translation, Brazilian Portuguese. Therefore, it was of interest to identify possible differences between words and phrases in the instrument, following the proposed model ^(^ ^7^ ^)^ . Thus, two versions emerged, namely: instrument 1 (INST1) and instrument 2 (INST2). The first with a more literal translation of the instrument and the second with a more interpretative translation, consistent with the language of the population. 

### Step II – Synthesis of the translations

The INST1 and INST2 versions were compared with the original instrument and synthesized, generating a single version, called instrument 12 (INST12). This step was carried out by a third translator, who had Portuguese as his mother tongue and was fluent in English.

### Step III – Back translation

The INST12 version obtained through the synthesis was translated into the original language of the scale, generating two versions: back translation 1 (BINST1) and back translation 2 (BINST2). This step was carried out by two bilingual translators whose mother tongue was the same as that of the original instrument (English). Both translators had no prior knowledge of the instrument and its use, also had no training in the area. With this process, the intention was to observe conceptual inaccuracies in the translation and ensure that the translated version was clear and precise regarding the content of the original version of the instrument.

### Step IV – Content review and validation by a committee of experts

 According to the translation framework used ^(^ ^7^ ^)^ , this phase is essential to achieve the transcultural translation of the instrument. The formation of a committee of experts is necessary to evaluate all versions obtained through the previous steps. 

 It is recommended that the number of expert participants on a committee be between five and ten ^(^ ^7^ ^-^ ^9^ ^)^ . The members of this committee were invited to participate based on the analysis of the curriculum on the *Lattes* Platform. The following filters were used as inclusion criteria for inviting experts: “Clinical Simulation” and “Validation Studies”. Those CVs that were updated in the year 2023 were extracted. Intentionally, experts were selected to compose the committee with, at least, 3 years of experience in the area of clinical simulation or in validation studies, experience in clinical practice, research or publications on the subject and expertise in conceptual structure in simulation. 

 The committee was made up of experts with experience in clinical simulation, scenario development and health education, who accepted the invitation. The objective was to evaluate all versions (INST1, INST2, INST12, BINST1 and BINST2), comparing them with the original instrument, to achieve semantic, idiomatic, experiential and conceptual equivalence between the original version of the instrument and the Portuguese version ^(^ ^7^ ^)^ . 

 Semantic equivalence determines whether there is parity in the meaning of words and grammatical representation. In idiomatic equivalence, there is an evaluation of expressions from the original language of the instrument, comparing them with those adapted from the instrument translated into Portuguese. In experiential equivalence, it is assessed whether the content described in the instrument is suitable for use in the target population. Finally, in conceptual equivalence, it was observed whether some words have similar meanings or whether they have the same importance in different languages and cultures ^(^ ^7^ ^)^ . 

 Then, this same group of experts carried out validation of the scale’s content validity. The following properties of psychometrics were considered: scope of the scale, clarity and relevance (or pertinence) of each item. In evaluating the scope of the instrument, the objective was to understand whether each domain or concept was adequately reached by the set of items presented. Regarding clarity, the focus was on understanding and writing the items, with the aim of evaluating whether each of them effectively expressed what was expected to be measured. Concerning relevance or pertinence, the importance of the items for achieving the objectives proposed with the application of the scale was assessed, and whether they reflected the concepts involved ^(^ ^7^ ^-^ ^8^ ^,^ ^10^ ^)^ . 

The experts filled out a document to measure the proportion of agreement between them on aspects of the instrument and its items, using an ordinal scale with a score from 1 to 4, with 1 = not relevant/clear/equivalent, 2 = item requires major revision to be relevant/clear/equivalent, 3 = item needs minor revision to be relevant/clear/equivalent and 4 = item relevant/clear/equivalent. Next, an assessment of the scale’s comprehensiveness was requested, which used a 4-point ordinal scale, with 1 = not comprehensive, 2 = scale needs major revision to be comprehensive, 3 = scale needs minor revision to be comprehensive and 4 = comprehensive scale.

Relevant notes about the items were evaluated by the study’s main researcher, who compiled the suggestions and sent them by email to the Committee of Experts for further online assessment of the translated version.

Therefore, at the end of this step, the pre-final version called Facilitator Competency Rubric – Brazilian Version (FCR-BV) was made available, which was consolidated by the consensus of ideas and opinions of experts.

### Step V – Pre-final version testing

 Testing the pre-final version was important for stabilizing the instrument, and is a step in which it is exposed to new evaluations ^(^ ^7^ ^)^ . 

 To carry out the test, health professionals who were facilitators of clinical simulation were invited, selected from the *Lattes* Platform, using the words “Clinical Simulation” as an inclusion criterion. In addition, simulation facilitators who were members of the *Sociedade Brasileira de Simulação na Saúde* (SOBRASSIM) were contacted. For both, it was requested to indicate individuals who could participate in this step of the study, using the snowball recruitment method ^(^ ^11^ ^)^ . 

 It is recommended that, for this process, the instrument should ideally be applied to a population of 30 to 40 individuals ^(^ ^7^ ^-^ ^8^ ^)^ . At least 30 professionals were then randomly selected to self-applying the instrument in the pre-final version. Professionals who accepted the invitation to participate in this step received the pre-final version of the Facilitator Competency Rubric - Brazilian Version for self-application; a characterization form about their professional profile, which included time of experience in simulation, sociodemographic and teaching characteristics; and a document in which they could share the clarity, understanding and ease of the instrument, completion time and a space for suggestions and changes. In the case of suggestions that could change the content of the item, it was again forwarded to the Committee of Experts for review for alignment and possible adaptation ^(^ ^8^ ^)^ . 

### Step VI – Submission and validation of the instrument by the authors

At this step, a report was sent to the author of the original instrument, containing the entire process of forming the final version, including previous versions originating from different translations.

### Validation step - Reliability test

 Reliability was assessed by analyzing the internal consistency and stability of the rubric. These individuals were approached in two ways: at an international event on realistic simulation and through the selection of the *Lattes* curriculum, as described in step V. Participants were invited to fill out a form on the Google Forms platform, with the same characterization instrument used in the pre-test phase and the final version of the FCR-BV instrument. Participants who agreed were contacted again after 15 days for a new application of the scale for test-retest evaluation ^(^ ^10^ ^,^ ^12^ ^-^ ^13^ ^)^ . 

### Analysis of results and statistics

 Equivalences and content validation were assessed quantitatively by calculating the Content Validity Index (CVI) and the modified Kappa Coefficient. Minimum values of 0.90 and 0.74, respectively, were considered acceptable ^(^ ^8^ ^,^ ^13^ ^)^ . For items that did not reach the established minimum score, experts’ suggestions were analyzed and incorporated into the instrument ^(^ ^8^ ^,^ ^14^ ^)^ . 

 The data obtained were tabulated in Microsoft Excel for Windows ^®^ spreadsheets, in which the measurements of the quantitative variables and the frequency measurements of the qualitative variables were calculated. The CVI was calculated by summing the agreements of the items classified as “3” or “4” by the experts and divided by the total number of responses to the instrument. Those items that received a score of “1” or “2” were reviewed by the committee. The modified Kappa Coefficient was obtained by the ratio of the proportion of times that experts agreed with the maximum proportion of times that they could agree ^(^ ^8^ ^,^ ^10^ ^,^ ^13^ ^)^ . 

 The scale’s reliability measurement was carried out by analyzing internal consistency, using Cronbach’s alpha Coefficient ^(^ ^10^ ^)^ . Values greater than 0.7 were considered to evaluate the consistency of the instrument ^(^ ^10^ ^,^ ^13^ ^)^ . 

 To evaluate the agreement between the measurements obtained in the test and retest in relation to the instrument scores, the Intraclass Correlation Coefficient was applied ^(^ ^10^ ^,^ ^13^ ^)^ . The scale was applied at two different times, with the aim of verifying whether the results obtained would be similar, that is, estimating whether there was consistency in the face of repeated measurements. It is expected that the facilitator’s competence will be the same at both times of application of the instrument ^(^ ^10^ ^,^ ^13^ ^)^ . Values greater than or equal to 0.70 were considered to indicate good reliability ^(^ ^8^ ^,^ ^10^ ^,^ ^13^ ^)^ . 

 Statistical Analysis Software ^®^ (SAS), version 9.4, was used in all analyses. 

### Ethical issues

To carry out the translation and cross-cultural adaptation, authorization was requested from the author of the original instrument and positive feedback regarding its use was received. The project was approved by the Research Ethics Committee, followed by all approval information (CAAE number: 68093623.2.0000.5374).

## Results

The first three steps went without difficulty and were carried out by experienced professionals in the field.

Regarding step IV of the study, around 16 experts were invited to participate in the Committee. Of these, only 12 returned contact. Two refused to participate, and ten experts accepted, however, only seven returned with the completed instrument and composed the committee of experts. This was formed exclusively by women, two working in the Northeast region of Brazil and the rest in the Southeast region, three post-doctors, two doctors, one master and one specialist, with an average of 14.42 years (standard deviation=3. 35) of education time in their areas. The committee had an average teaching time of around 8 years (standard deviation=5.44), 6.57 years (standard deviation=3.40) working with clinical simulation and with a total of 21 articles published on the theme.

 Two rounds of analysis were carried out by the committee of experts, one before the final pre-test of the available version and another after the final pre-test. First, the CVI and modified Kappa values were calculated for all instrument items, as shown in Table 1 . 

**Table 1:** - Content Validity Index (CVI ^*^ ) results obtained in the first round of expert evaluation. Campinas, SP, Brazil, 2023

	**Equivalences**								**Clarity**		**Relevance**	
**Item - Domain**	**Semantic**		**Idiomatic**		**Conceptual**		**Cultural**					
	**CVI** ^*^	***Kappa***	**CVI** ^*^	***Kappa***	**CVI** ^*^	***Kappa***	**CVI** ^*^	***Kappa***	**CVI** ^*^	***Kappa***	**CVI** ^*^	***Kappa***
**1.2 - Domain 1**	0.857	0.857	0.893	0.893	0.893	0.893	0.929	0.928	0.893	0.893	1.00	1.00
**1.4 - Domain 1**	0.857	0.857	0.893	0.893	0.929	0.928	0.929	0.929	0.964	0.964	0.857	0.857
**1.5 - Domain 1**	0.893	0.893	0.929	0.929	0.964	0.964	0.964	0.964	0.929	0.929	0.964	0.964
**1.6 - Domain 1**	0.857	0.857	0.929	0.929	1.00	1.00	1.00	1.00	1.00	1.00	1.00	1.00
**1.1 - Domain 2**	0.893	0.893	0.964	0.964	0.964	0.964	0.964	0.964	0.964	0.964	0.964	0.964
**1.2 - Domain 3**	0.893	0.893	0.929	0.929	0.964	0.964	0.964	0.964	0.964	0.964	0.964	0.964
**1.6 - Domain 4**	0.893	0.893	0.929	0.929	0.964	0.964	0.964	0.964	0.929	0.929	1.00	1.00

* CVI = Content Validity Index

 Eleven items (1.1, 1.3, 1.7 from Domain 1; 1.3 from Domain 2; 1.3, 1.5 from Domain 3; 1.2, 1.4, 1.8 from Domain 4; 1.1, 1.2 from Domain 5) do not appear in Table 1 because, despite having had acceptable CVI and modified Kappa values, they received grammatical suggestions and underwent specific reformulations. 

 Despite there being no CVI below 0.9, the item that describes the facilitator’s classification concepts received a suggestion that was accepted as a modification proposal. The classification “experienced beginner” in the synthesis version was replaced by “advanced beginner”, as suggested by the experts. In Figure 2 , all the changes made by the committee when evaluating the synthesis version are detailed. 

 To test the pre-final version in step V of the study, 134 invitations were sent to experts selected by the *Lattes* Platform, and only 33 professionals agreed to participate. Of these participants, 66.7% (22) were female, and 51.5% (17) had a doctorate as their highest level of education. Regarding graduation, 63.6% (21) were nurses, 27.3% (9) were doctors and the remainder were veterinarian (1), physiotherapist (1) and pedagogue (1). The sample of simulation facilitators had an average of 19.5 years of education in their areas (standard deviation=12.91) and 14.2 years of teaching time (standard deviation=12.11). All had an average of 6.7 years (standard deviation=4.08) of experience with clinical simulation. The average time spent answering the instrument was 11.6 minutes (standard deviation=8.31). All participants considered the instrument adequate or partially adequate to assess the facilitator’s competence in simulation, and found the items understandable or partially understandable. A space was opened for suggestions, which were subsequently taken to the committee of experts in a second round of evaluation, for analysis and incorporation of proposals made. 10 proposals were made, however, only two were accepted by the committee. None of the proposals affected the content of the items. Figure 3 shows the changes made in this second round of instrument evaluation. 

**Figure 2:** - Synthesis version and result version of the committee of experts evaluation. Campinas, SP, Brazil, 2023

**Item**	**Synthesis version**	**Version resulting from the evaluation of the committee of experts**
Concepts	Novice (1) to experienced beginner (2)	Novice (1) to advanced beginner (2)
1.1 Domain 1- Programming	- Can identify the need for small groups at the bedside. - Has creativity in planning activities. - Can program the learning experience in such a way that it is optimal.	- Identifies the need for small groups at the bedside. - Demonstrates creativity in planning activities. - Schedules the learning experience in such a way that it is optimal.
1.2 Domain 1 - Learning objectives	- Covers the cognitive, affective and psychomotor learning domains. - Correlates the objectives of all domains to the education or experience of the participants.	- Addresses the cognitive, affective and psychomotor learning domains. - Correlates the objectives of all domains to the educational level or experience of the participants.
1.3 Domain 1 - Planning process	- Informs laboratory team that a simulation will be conducted. - Works together with the laboratory team to fully achieve learning objectives. - Analyzes previous clinical simulations, aiming to improve the learning experience.	- Informs laboratory team of plans to conduct the simulation. - Collaborates with the laboratory team to fully achieve learning objectives. - Reviews previous clinical simulations to enhance the learning experience.
1.4 Domain 1 - Fidelity level (e.g., environment, simulation modality)	- Plans a level of fidelity that encompasses the desired outcomes.	- Plans the level of fidelity that encompasses the desired results.
1.5 Domain 1 - Availability of supplies/equipment	- Lists the supplies and equipment needed in the simulation - Organizes necessary learning materials by priority. - Develops or improves materials to stimulate critical thinking in learners.	- Lists the supplies and equipment needed for the simulation. - Organizes teaching materials by priority. - Develops or improves materials to stimulate critical thinking in participants.
1.6 Domain 1 - Preparation requirements	- Informs participants of what is necessary to prepare before the simulation. - Can assess whether participants are prepared for the simulation.	- Informs participants about what is necessary to prepare before the simulation. - Determines whether participants are prepared for the simulation.
1.7 Domain 1 - Assessment methods	- Tries to use good psychometric assessment tools.	- Plans to use assessment tools with good psychometric properties.
1.3 Domain 2 - Role identification	- Analyzes which role should be given to which participant to optimize learning, considering the strengths and weaknesses already identified.	- Analyzes which role should be given to each participant to optimize learning, considering the strengths and weaknesses already identified.
1.3 Domain 3 - Participants involvement	- During the simulation, gives good instructions or incentives for all participants to get involved. - Employs different methods to engage those who are not participating much.	- Provides clues or incentives for all participants to engage during the simulation. - Employs different methods to engage those who are not participating much in the simulation.
1.5 Domain 3 - Time/duration	- Continues the written scenario, regardless of time management. - Adapts throughout the experience, seeking to cover all learning objectives within the allocated time.	- Continues the scenario according to the script, without worrying about time management. - Adapts throughout the experience, seeking to cover all learning objectives within the established time.
1.2 Domain 4 - Facilitates reflection	- Stimulates in-depth analysis of choice processes and higher-order thinking.	- Stimulates in-depth analysis of decision-making processes, critical thinking and problem solving.
1.4 Domain 4 - Active listening	- In the discussion, contributes more than the participants themselves.	- Contributes more than the participants themselves during the discussion.
1.6 Domain 4 - Learning Objectives	- Pays attention to the events in the scenario. - Can assess whether the learning objectives were assimilated. - Assists participants in assessing the level of assimilation of learning objectives.	- Focus their attention on the events in the scenario. - Can assess whether the learning objectives were achieved. - Helps participants determine the achievement of learning objectives.
1.8 Domain 4 - Summary	- Helps participants to summarize the simulation.	- Supports participants as they summarize the simulation.
1.1 Domain 5 - Experience	- Uses designed methods to collect data from participants, team, and teaching staff about the simulation.	- Uses structured methods to collect data from participants, team, and teaching staff about the simulation.
1.2 Domain 5 - Participants	- Uses designed methods to collect data about participants and learning.	- Uses structured methods to collect data about participants and learning.

**Figure 3:** - Pre-final version and final version resulting from the 2 ^nd^ evaluation of the committee of experts. Campinas, SP, Brazil, 2023

**Item**	**Pre-final version**	**Version resulting from the 2nd evaluation of the committee of experts (Final version)**
Introductory text	No content	Dear, if you are carrying out your self-assessment or evaluating a professional using the Brazilian version of the Facilitator Competency Rubric (FCR), we inform you that the competencies for a facilitator are evaluated through five domains (preparation, prebriefing, facilitation, debriefing and evaluation). For each domain, there is a list of items. We recommend that you read each item carefully and choose the rating that best represents you or the professional you are evaluating: **(1) Beginner; (2) Advanced Beginner; (3)** **Competent; (4) Proficient; (5)** **Expert** At the end, you will find the expected score for each domain.
Score line in each domain	Template for the total of the three columns — “Preparation” Section 0-14 = “Beginner” to “Advanced Beginner” (needs supervision from a “Proficient” to “Expert” facilitator). 15-27 = “Competent”. 28-35 = “Proficient” to “Expert” (able to supervise a “Beginner” to “Advanced Beginner” facilitator).	Template for the total of the three columns — “Preparation” Section 0-14 = “Beginner” to “Advanced Beginner” (needs supervision from a “Proficient” to “Expert” facilitator). 15-27 = “Competent”. 28-35 = “Proficient” to “Expert” (able to supervise a “Beginner” to “Advanced Beginner” facilitator).

 In Figure 3 , it can be seen that the inclusion of an introductory text was proposed to guide the facilitator on filling out the instrument, which achieved 100% agreement between the evaluators. 

At the end of all steps, the final version of the Facilitator Competency Rubric - Brazilian version (FCR-BV) was sent to the author of the original instrument, who approved the adaptation process.

 Regarding the FCR-BV reliability measurement, 52 simulation facilitators self-completed the instrument. Of these participants, 78.85% (41) were female, 73.1% (38) were nurses, 17.3% (9) were doctors, 5.77% (3) were pharmacists, 1.93% (1) were nutritionists and 1.93% (1) were biologists. Around 38.5% (20) of these professionals had a PhD as their highest level of training, 25% (13) Specialization, 23% (12) Master and 13.5% (7) Post-Doctorate. Furthermore, the professionals had an average of 17.19 years of education time (standard deviation=8.24), with an average of 10.98 years of experience in teaching (standard deviation=6.61), and they had used simulation in their teaching/work practice for an average of 6.4 years (standard deviation=5.28). Table 2 presents the classification of facilitators in simulation and the results of the reliability measures of the Brazilian version of the FCR, verified based on Cronbach’s alpha data, measured for each domain and for the general scale, and the values of the Intraclass Correlation Coefficient (ICC). 

**Table 2:** - Classification of participants in the test of the pre-final and final versions and reliability of the Brazilian version of the Facilitator Competency Rubric. Campinas, SP, Brazil, 2023

**Scale domains**	**Classification**	**Pre-final version testing (n=33)**	**Reliability Test** **(n=52)**	**Cronbach’s Alpha** **(n=52)**	**ICC** ^*^ **(n=15)**
**Preparation**		Average ^†^ =24.54 (SD ^‡^ =7.30)	Average ^†^ =25.11 (SD ^‡^ =6.25)	0.911	0.95
	Beginner to Advanced Beginner	12.1% (4)	3.8% (2)		
	Competent	51.5% (17)	57.7% (30)		
	Proficient to Expert	36.4% (12)	38.5% (20)		
**Prebriefing**		Average ^†^ =14.69 (SD ^‡^ =3.99)	Average ^†^ =13.82 (SD ^‡^ =4.26)	0.876	0.96
	Beginner to Advanced Beginner	9.1% (3)	9.6% (5)		
	Competent	42.4% (14)	55.8% (29)		
	Proficient to Expert	48.5% (16)	34.6% (18)		
**Facilitation**		Average ^†^ =22.42 (SD ^‡^ =5,89)	Average ^†^ =22.82 (SD ^‡^ =4.93)	0.906	0.95
	Beginner to Advanced Beginner	12.1% (4)	1.9% (1)		
	Competent	27.3% (9)	53.8% (28)		
	Proficient to Expert	60.6% (20)	44.3% (23)		
**Debriefing**		Average ^†^ =30.51 (SD ^‡^ =7.45)	Average ^†^ =29.56 (SD ^‡^ =7.08)	0.947	0.97
	Novice to Advanced Beginner	6.1% (2)	5.8% (3)		
	Competent	39.4% (13)	55.7% (29)		
	Proficient to Expert	54.5% (18)	38.5% (20)		
**Evaluation**		Average ^†^ =14.06 (SD ^‡^ =3.85)	Average ^†^ =13.76 (SD ^‡^ =3.57)	0.878	0.97
	Novice to Advanced Beginner	9.1% (3)	9.6% (5)		
	Competent	48.5% (16)	61.5% (32)		
	Proficient to Expert	42.4% (14)	28.9% (15)		
				Total=0.980	-

* ICC = Intraclass correlation coefficient;

† Average score per domain;

‡ SD = Standard deviation per domain

In the scale stability test, 35 of these participants agreed to contribute to the study, however, only 15 returned with the completed instrument after 15 days of self-application.

## Discussion

The Brazilian version of the Facilitator Competency Rubric and its adaptation process were successful in different areas, in addition to presenting reliable measures of internal consistency and content validity.

 The adoption of a theoretical and methodological framework for the process of cultural adaptation of an instrument is directly associated with the quality of the result obtained after all the steps carried out. The rigor and complexity required to make items equivalent between the original and adapted versions of an instrument are fundamental to support the qualified use of this material. Ensuring equivalence between the two versions begins by choosing an appropriate methodology ^(^ ^7^ ^,^ ^15^ ^)^ . This entire process was followed in the cultural adaptation of the FCR to the Brazilian context in this study. 

 Studies that compared cultural adaptation methods and their validations showed that the back translation step is not mandatory, as there is no significant difference in the final result of the instrument. However, this step can be useful as a communication tool with the author of the original instrument ^(^ ^16^ ^-^ ^17^ ^)^ . Furthermore, the effective participation of the Committee of Experts is essential to guarantee equivalences, and the constant return of the notes generated by the pre-final version testing step to this multidisciplinary team allows for the accuracy of the content of the items in the comparison between the original and translated versions of the instrument ^(^ ^16^ ^-^ ^17^ ^)^ . In this study, these two steps were completed, as provided for in the chosen methodological framework ^(^ ^7^ ^)^ . 

 It should be noted that, for the composition of the committee of experts, participant profiles were sought that suited what is recommended in the literature ^(^ ^7^ ^-^ ^8^ ^)^ . A multidisciplinary group working on different fronts in simulation and validation studies reinforces the quality and care that this study took in ensuring better validation of the instrument’s content. Moreover, choosing a CVI value of 0.9 increases the safety and rigor of the process, as seen in other studies ^(^ ^18^ ^-^ ^20^ ^)^ . 

 The author of the original instrument presented in her study that the FCR was designed to be used in observation of the simulation facilitator, which makes teaching staff more reluctant to be evaluated while exercising their role as teachers. However, the results show that the self-application of the instrument provides more credibility to the findings and guides the development of the teaching staff ^(^ ^5^ ^)^ . Therefore, the present study chose the strategy of self-application of the instrument to obtain the results. 

 Another important choice in the FCR-BV validation process was the inclusion of participants with experience in clinical simulation and a text that guides its completion. The choice to include only people with experience in using simulation was based on the German translation study of the instrument, in which the authors highlighted that the inclusion of participants with little or no training in simulation may have interfered with the results achieved ^(^ ^21^ ^)^ . This observation reveals that the individual who fills out the instrument needs to have a minimum of knowledge about clinical simulation and the domains that the questionnaire aims to evaluate. 

Furthermore, the inclusion of the guidance text did not directly interfere with the content of the original instrument, and was also suggested by the research participants. Without a doubt, the availability of a guide is essential to assist the professional who applies the teaching strategy in understanding the objectives to evaluate themselves as a facilitator.

 The professionals who participated in step V of the study presented pertinent suggestions regarding the reality of the topic. This reinforces what the literature shares as important: the inclusion of the target audience in data collection and their contributions to improving the understanding of the instrument, and facilitating adherence to its use within practice and obtaining information and results that are truthful and of quality ^(^ ^18^ ^-^ ^19^ ^)^ . 

 In the present study, although there are no previous reports on the time taken to complete the FCR inside or outside the simulation scenario ^(^ ^5^ ^,^ ^21^ ^)^ , there was a great disparity in the recording of time to complete the instrument. There was a variation of 2 to 40 minutes in time to complete the scale, and comments were made by the facilitators about the large number of items (29) present in the instrument, which may be associated with the greater amount of time to complete the self-assessment. 

 Regarding the analysis of internal consistency, the results from Brazil are similar to those from the German version ^(^ ^21^ ^)^ , with values above 0.8 for all domains. It was not possible to compare with the values found in the original study, since the measurement of internal consistency was carried out differently, using the G Coefficient, which showed good to excellent results ^(^ ^5^ ^)^ , also verified in the Brazilian version. 

 To measure the stability of the scale, verified through test-retest, the three versions presented ICC above the reference values of 0.70 ^(^ ^14^ ^-^ ^15^ ^)^ , highlighting how stable the measures that the instrument assesses at different times are. 

 This study was not without limitations. In the testing step of the pre-final version, it was not possible to calculate the response rate, as snowball recruitment was adopted for self-completion of the instrument, which can be considered a sampling bias. Besides, the number of individuals who participated in the test-retest step to measure reliability was lower than that recommended in the literature, which provides for a minimum of 50 individuals ^(^ ^8^ ^,^ ^13^ ^-^ ^14^ ^)^ . We recommend that, in future studies with a larger sample size, evaluations of the scale structure be carried out, through exploratory factor analysis, followed by confirmatory factor analysis, to confirm the arrangement of the items in the instrument domains. 

Providing the Brazilian version of the FCR with the properties of tested and validated measures for the academic and institutional context contributes to the advancement of good clinical simulation practices in scopes of practice increasingly used in the health area. Using the rubric, the facilitator will be able to recognize their strengths and limitations when applying the simulation methodology, that is, the Brazilian version of the FCR can be used in the development of professionals as facilitators in clinical simulation, by scoring the skills that must still be developed. With this, professionals who intend to act in the role of facilitators in an effective and efficient way will be able to seek training more relevant to their objectives to promote better teaching and learning conditions, using all the potential that clinical simulation offers. We also cannot exclude the advantages of advancing science in the field of simulation research with the use of this instrument.

## Conclusion

The process of translating and adapting the FCR carefully followed the steps described as a reference in international literature. The inclusion of this instrument in the national scenario of Brazilian clinical simulation can increase knowledge about facilitator skills and the development of new skills for professionals who use this teaching strategy. The Brazilian version of the FCR proved to be suitable for use and self-assessment. Studies with a larger sample size and evaluating different measurement properties are recommended to increase the precision and validity of the instrument.
