# Supplementary material for: Cultural adaptation and evaluation of the measurement properties of the Facilitator Competency Rubric for clinical simulation facilitators
Source: Rev Lat Am Enfermagem. 2024 Jul 29;32:e4257. doi: 10.1590/1518-8345.7214.4257 (PMC11295262; doi:10.1590/1518-8345.7214.4257)
Supplement: Translation

# Translation: Adaptación cultural y evaluación de las propiedades de medición de la *Facilitator Competency Rubric* para facilitadores de simulación clínica

**DOI:** 10.1590/1518-8345.7214.4256

**Authors:** Daniel Gonçalves Campos, Juliany Lino Gomes Silva, Ruana Luiz Ferreira da Silva, Angélica Olivetto Almeida, Ana Luísa Brandão de Carvalho Lira, Ana Railka de Souza Oliveira-Kumakura

**Affiliations:** 1 Universidade Estadual de Campinas, Faculdade de Enfermagem, Campinas, SP, Brasil.; 2 Universidade Federal do Ceará, Departamento de Enfermagem, Fortaleza, CE, Brasil.; 3 Université Paris Cité, Département Universitaire en Sciences Infirmières, Paris, Francia.

**Keywords:** Entrenamiento Simulado, Encuestas y Cuestionarios, Educación, Enseñanza, Docentes, Competencia Profesional

## Abstract

**Objetivo:**

traducir y adaptar la *Facilitator Competency Rubric* a la lengua portuguesa y a la cultura brasileña, y analizar las propiedades de medición.

**Método:**

estudio metodológico que completó las etapas de traducción, síntesis de las traducciones, *back translation* , revisión por un Comité de Expertos compuesto por 7 profesionales, prueba de la versión pre-final con 33 facilitadores de simulación y presentación a la autora del instrumento original. Para la validación de contenido se calculó el Índice de Validez de Contenido y el Coeficiente Kappa modificado. Para determinar la confiabilidad, 52 y 15 facilitadores de simulación evaluaron el α de Cronbach y el Coeficiente de Correlación Intraclase, respectivamente.

**Resultados:**

se realizaron dos rondas de evaluación de contenidos, con modificaciones de 19 ítems en la primera evaluación y 3 ítems en la segunda. La escala general alcanzó α de Cronbach de 0,98 y Coeficientes de Correlación Intraclase de 0,95 a 0,97.

**Conclusión:**

la *Facilitator Competency Rubric* fue traducida y adaptada culturalmente a la realidad brasileña y presentó validez de contenido, confiabilidad y estabilidad, con resultados seguros para su uso en la enseñanza y la investigación.

## Destacados

**(1)** La versión brasileña de la FCR tiene contenido válido y es confiable para uso nacional.

**(2)** La FCR adaptada al contexto brasileño mide la competencia del facilitador de la simulación.

**(3)** Medir la competencia con la FCR favorece el escenario de la capacitación docente en simulación.

## Introducción

 El aprendizaje basado en simulación ha tenido un auge en los últimos años y ha sido una estrategia de enseñanza ampliamente utilizada por programas de grado en diferentes áreas de la salud ^(^ ^1^ ^-^ ^2^ ^)^ . Los investigadores señalan que aproximadamente la mitad de las horas referidas a la experiencia clínica tradicional pueden ser reemplazadas por experiencias clínicas simuladas si se cumplen ciertas condiciones para una buena ejecución, como una formación formal en pedagogía de simulación, un número adecuado de profesores para ayudar a los estudiantes, la presencia de especialistas en realizar un *debriefing* basado en evidencia y recursos materiales adecuados para que el ambiente sea lo más realista posible ^(^ ^2^ ^)^ . 

 Para ello, a lo largo de los años se han desarrollado diferentes instrumentos de medición para evaluar elementos necesarios para la implementación de esta estrategia docente o para evaluar sus resultados, especialmente los de los participantes ^(^ ^3^ ^)^ . Por ejemplo, la *National League for Nursing* (NLN) y la *International Nursing Association for Clinical and Simulation Learning* (INACSL) proporcionan instrumentos que han sido traducidos y validados para diferentes culturas y que se enfocan en evaluar el desempeño de habilidades, la satisfacción, la percepción de la experiencia educativa, el conocimiento/aprendizaje, el pensamiento crítico/juicio clínico, la autoconfianza/autoeficacia, el *debriefing* , la competencia del facilitador, y en evaluar el nivel de organización respecto al diseño del escenario simulado ^(^ ^4^ ^)^ . 

 La mayoría de estos instrumentos evalúan la experiencia del estudiante con la simulación, sin embargo, la evaluación del individuo que facilita la simulación aún está indefinida, a pesar de que se incluyen evidencias de su desempeño en otro tipo de herramientas ^(^ ^5^ ^)^ . Entre las escalas que se centran en el facilitador se encuentra la *Facilitator Competency Rubric* (FCR). Fue desarrollada para evaluar y medir el nivel de competencia del educador para facilitar el aprendizaje mediante simulación, y presenta versiones en inglés y en alemán. Se utiliza el marco teórico de Benner para diferenciar los niveles de competencia del facilitador, representados por: principiante, principiante avanzado, competente, eficiente y experto ^(^ ^5^ ^)^ . 

 El facilitador es considerado el educador profesional que es responsable de monitorear y garantizar toda la experiencia basada en simulación de la manera más segura posible para que los estudiantes trabajen de forma cohesiva, enfocándose en comprender los objetivos de aprendizaje y desarrollar un plan para lograr los resultados deseados. Ser facilitador requiere capacitación y habilidades para liderar, apoyar y encontrar formas de ayudar a los alumnos a lograr los resultados esperados ^(^ ^6^ ^)^ . 

 El facilitador principiante es una persona inexperta cuyos comportamientos y decisiones se guían por reglas preestablecidas y que puede aprender las tareas, pero no sabe qué hacer con la información obtenida. El principiante avanzado presenta un desempeño aceptable. El competente es capaz de desarrollar, implementar y priorizar una estrategia de acción para resolver un problema, sintiéndose en control de la situación. El facilitador eficiente es capaz de ver la situación desde una perspectiva más global basado en su experiencia, actuando con mayor rapidez y flexibilidad que el competente. Por último, el experto no se basa en reglas, sino en su experiencia e intuición. Dentro del entorno de educación y enseñanza, los profesionales pueden avanzar a través de estos niveles a medida que cambian responsabilidades laborales o aprenden nuevas estrategias de enseñanza ^(^ ^5^ ^)^ . 

 El instrumento FCR se compone de cinco dimensiones: preparación, *prebriefing* , facilitación, *debriefing* y evaluación. Para estas dimensiones hay un total de 29 ítems, que se pueden clasificar en una escala ordinal desde principiante (1) hasta experto (5). El estudio de desarrollo de la escala encontró que la confiabilidad entre evaluadores era buena. Los coeficientes de Generalización (G), utilizados para evaluar la confiabilidad entre evaluadores y determinar la cantidad de varianza atribuible a ellos, variaron de bueno a excelente (0,80 a 0,99), y los ítems plazo, día de la semana, hora del día y tipos de simulación fueron predictores significativos de la puntuación global del instrumento ^(^ ^5^ ^)^ . 

 Desde la perspectiva de que la FCR es un instrumento que, en el escenario brasileño, puede orientar para la capacitación del profesorado y ser utilizado como herramienta de autoevaluación o de evaluación formativa o sumativa de un facilitador de simulación ^(^ ^5^ ^)^ , se propone realizar este estudio. El objetivo fue traducir y adaptar la *Facilitator Competency Rubric* a la lengua portuguesa y a la cultura brasileña, y analizar las propiedades de medición. 

## Método

### Diseño del estudio

 Estudio metodológico, que completó las siguientes etapas: traducción, síntesis de las traducciones, *back translation* , revisión por un comité de expertos, prueba de la versión final y presentación a la autora del instrumento original ^(^ ^7^ ^)^ . Además, se realizó validación de contenido y medición de confiabilidad. La investigación fue descrita con base en los criterios adoptados por el *checklist* del *COnsensus-Based Standards for the selection of health Measurement INstruments* (COSMIN) ^(^ ^8^ ^)^ . La Figura 1 aclara el flujograma metodológico del estudio. 

**Figura 1:** - Flujograma metodológico del estudio. Campinas, SP, Brasil, 2023

## Período

El período de recolección de datos se desarrolló después de la aprobación del estudio por el Comité de Ética en Investigación y se realizó entre abril y septiembre de 2023.

### Etapa I – Traducciones

 El instrumento fue traducido del inglés al portugués brasileño, obteniendo dos versiones iniciales: una realizada por un traductor con conocimiento de terminologías de simulación y salud y la otra por un traductor sin conocimientos en el área. Ambos profesionales dominan el idioma original del instrumento y son originarios de Brasil, con experiencia en el idioma de destino de la traducción, el portugués brasileño. Por lo tanto, fue de interés identificar posibles diferencias entre palabras y frases en el instrumento, siguiendo el modelo propuesto ^(^ ^7^ ^)^ . Surgieron así dos versiones, a saber: instrumento 1 (INST1) e instrumento 2 (INST2). La primera con una traducción más literal del instrumento y la segunda con una traducción más interpretativa, acorde con el idioma de la población. 

### Etapa II – Síntesis de las traducciones

Las versiones INST1 e INST2 fueron comparadas con el instrumento original y sintetizadas, generando una única versión, denominada instrumento 12 (INST12). Esta etapa estuvo a cargo de un tercer traductor, que tenía como lengua materna el portugués y dominaba el inglés.

### Etapa III – *Back translation*

 La versión INST12 obtenida a través de la síntesis fue traducida al idioma original de la escala, generándose dos versiones: *back translation* 1 (BINST1) y *back translation* 2 (BINST2). Esta etapa fue realizada por dos traductores bilingües cuya lengua materna era la misma que la del instrumento original (inglés). Ambos traductores no tenían conocimiento previo del instrumento y su uso, y no tenían capacitación en el área. Con este proceso se pretendió observar imprecisiones conceptuales en la traducción y asegurar que la versión traducida fuera clara y precisa respecto del contenido de la versión original del instrumento. 

### Etapa IV – Revisión y validación de contenidos por un comité de expertos

 Según el marco de traducción utilizado ^(^ ^7^ ^)^ , esta fase es fundamental para lograr la traducción transcultural del instrumento. Es necesaria la conformación de un comité de expertos para evaluar todas las versiones obtenidas a través de las etapas anteriores. 

 Se recomienda que el número de expertos participantes en un comité sea entre cinco y diez ^(^ ^7^ ^-^ ^9^ ^)^ . Los integrantes de este comité fueron invitados a participar a partir del análisis del currículum en la Plataforma *Lattes* . Se utilizaron como criterios de inclusión para invitar a expertos los siguientes filtros: “Simulación Clínica” y “Estudios de Validación”. Se extrajeron aquellos CV que fueron actualizados en el año 2023. Intencionalmente, se seleccionaron expertos para formar el comité con, al menos, 3 años de experiencia en el área de simulación clínica o en estudios de validación, experiencia en la práctica clínica, investigaciones o publicaciones sobre el tema y experiencia en estructura conceptual en simulación. 

 El comité estuvo integrado por expertos con experiencia en simulación clínica, desarrollo de escenarios y educación para la salud, quienes aceptaron la invitación. El objetivo fue evaluar todas las versiones (INST1, INST2, INST12, BINST1 y BINST2), comparándolas con el instrumento original, para lograr equivalencia semántica, idiomática, experiencial y conceptual entre la versión original del instrumento y la versión en portugués ^(^ ^7^ ^)^ . 

 La equivalencia semántica determina si existe paridad en el significado de las palabras y la representación gramatical. En la equivalencia idiomática se evalúan expresiones del idioma original del instrumento, comparándolas con aquellas adaptadas del instrumento traducido al portugués. En la equivalencia experiencial se evalúa si el contenido descrito en el instrumento es adecuado para su uso en la población objetivo. Por último, en la equivalencia conceptual, se observó si algunas palabras tienen significados similares o si tienen la misma importancia en diferentes idiomas y culturas ^(^ ^7^ ^)^ . 

 Luego, este mismo grupo de expertos realizó la validación del contenido de la escala. Se consideraron las siguientes propiedades de la psicometría: alcance de la escala, claridad y relevancia (o pertinencia) de cada ítem. Al evaluar el alcance del instrumento, el objetivo fue comprender si cada dominio o concepto fue alcanzado adecuadamente por el conjunto de ítems presentados. En cuanto a la claridad, el foco estuvo en la comprensión y redacción de los ítems, con el objetivo de evaluar si cada uno de ellos expresaba efectivamente lo que se esperaba medir. Respecto a la relevancia o pertinencia, se valoró la importancia de los ítems para la consecución de los objetivos propuestos con la aplicación de la escala, y si reflejaban los conceptos involucrados ^(^ ^7^ ^-^ ^8^ ^,^ ^10^ ^)^ . 

Los expertos llenaron un documento para medir la proporción de acuerdo entre ellos sobre aspectos del instrumento y sus ítems, utilizando una escala ordinal con puntuación de 1 a 4, siendo 1 = no relevante/claro/equivalente, 2 = ítem requiere mayor revisión para que sea relevante/claro/equivalente, 3 = el ítem necesita una revisión menor para ser relevante/claro/equivalente y 4 = ítem relevante/claro/equivalente. A continuación, se solicitó una evaluación del alcance de la escala, la cual utilizó una escala ordinal de 4 puntos, siendo 1 = no integral, 2 = la escala necesita una revisión importante para ser integral, 3 = la escala necesita una revisión menor para ser integral y 4 = escala integral.

 El investigador principal del estudio evaluó las notas relevantes sobre los ítems, recopiló las sugerencias y las envió por correo electrónico al Comité de Expertos para una evaluación adicional *on line* de la versión traducida. 

 Por lo tanto, al final de esta etapa, se puso a disposición la versión pre-final denominada *Facilitator Competency Rubric – Brazilian Version* (FCR-BV), que fue consolidada por el consenso de ideas y opiniones de los expertos. 

### Etapa V – Prueba de la versión pre-final

 La prueba de la versión pre-final fue importante para estabilizar el instrumento, y es una etapa en la que se lo expone a nuevas evaluaciones ^(^ ^7^ ^)^ . 

 Para la realización de la prueba se invitó a profesionales de la salud facilitadores de la simulación clínica, seleccionados de la Plataforma *Lattes* , utilizando como criterio de inclusión las palabras “Simulación Clínica”. Además, se contactó a los facilitadores de la simulación que eran miembros de la *Sociedade Brasileira de Simulação na Saúde* (SOBRASSIM). Para ambos se solicitó indicar individuos que podrían participar en esta etapa del estudio, utilizando el método de reclutamiento de bola de nieve ^(^ ^11^ ^)^ . 

 Se recomienda que, para este proceso, el instrumento sea aplicado idealmente a una población de 30 a 40 individuos ^(^ ^7^ ^-^ ^8^ ^)^ . Luego, se seleccionaron aleatoriamente al menos 30 profesionales para autoadministrarse el instrumento en la versión pre-final. Los profesionales que aceptaron la invitación a participar de esta etapa recibieron la versión pre-final de la *Facilitator Competency Rubric -* *Brazilian Version* para autoaplicación; una ficha de caracterización de su perfil profesional, que incluía tiempo de experiencia en simulación, características sociodemográficas y docentes; y un documento en el que pudieran compartir sobre la claridad, comprensión y facilidad del instrumento, tiempo de finalización y un espacio para sugerencias y cambios. En el caso de sugerencias que pudieran cambiar el contenido del ítem, se remitió nuevamente al comité de expertos para su revisión para alineación y posible adaptación ^(^ ^8^ ^)^ . 

### Etapa VI – Presentación y validación del instrumento por parte de los autores

En esta etapa, se envió un informe a la autora del instrumento original, que contiene todo el proceso de formación de la versión final, incluyendo las versiones anteriores provenientes de diferentes traducciones.

### Etapa de validación - Prueba de confiabilidad

 La confiabilidad se evaluó analizando la consistencia interna y la estabilidad de la rúbrica. Se contactó a estos individuos de dos maneras: en un evento internacional sobre simulación realista y mediante la selección del currículo *Lattes* , como se describe en la etapa V. Los participantes fueron invitados a completar un formulario en la plataforma *Google Forms* , con el mismo instrumento de caracterización utilizado en la fase de la prueba previa y versión final del instrumento FCR-VB. Los participantes que aceptaron fueron contactados nuevamente después de 15 días para una nueva aplicación de la escala de evaluación prueba-reprueba ^(^ ^10^ ^,^ ^12^ ^-^ ^13^ ^)^ . 

### Análisis de los resultados y estadística

 Las equivalencias y la validación de contenido se evaluaron cuantitativamente mediante el cálculo del Índice de Validez de Contenido (IVC) y del Coeficiente Kappa modificado. Se consideraron aceptables valores mínimos de 0,90 y 0,74, respectivamente ^(^ ^8^ ^,^ ^13^ ^)^ . Para los ítems que no alcanzaron la puntuación mínima establecida, se analizaron e incorporaron al instrumento sugerencias de los expertos ^(^ ^8^ ^,^ ^14^ ^)^ . 

 Los datos obtenidos fueron tabulados en hojas de cálculo del *Microsoft Excel for Windows* ^®^ , en las que se calcularon las mediciones de las variables cuantitativas y las mediciones de frecuencia de las variables cualitativas. El IVC se calculó sumando los acuerdos de los ítems clasificados como “3” o “4” por los expertos y dividido por el número total de respuestas al instrumento. Aquellos ítems que recibieron una puntuación de “1” o “2” fueron revisados por el Comité. El Coeficiente Kappa modificado se obtuvo por la relación entre la proporción de veces que los expertos estuvieron de acuerdo con la proporción máxima de veces que pudieron estar de acuerdo ^(^ ^8^ ^,^ ^10^ ^,^ ^13^ ^)^ . 

 La medición de la confiabilidad de la escala se realizó analizando la consistencia interna, utilizando el Coeficiente alfa de Cronbach ^(^ ^10^ ^)^ . Se consideraron valores mayores a 0,7 para evaluar la consistencia del instrumento ^(^ ^10^ ^,^ ^13^ ^)^ . 

 Para evaluar la concordancia entre las mediciones obtenidas en la prueba y reprueba con relación a las puntuaciones del instrumento, se aplicó el Coeficiente de Correlación Intraclase ^(^ ^10^ ^,^ ^13^ ^)^ . La escala se aplicó en dos momentos diferentes, con el objetivo de verificar si los resultados obtenidos serían similares, es decir, estimar si había consistencia ante mediciones repetidas. Se espera que la competencia del facilitador sea la misma en ambos momentos de aplicación del instrumento ^(^ ^10^ ^,^ ^13^ ^)^ . Se consideró que valores mayores o iguales a 0,70 indicaban buena confiabilidad ^(^ ^8^ ^,^ ^10^ ^,^ ^13^ ^)^ . 

 En todos los análisis se utilizó el *Statistical Analysis Software* ^®^ (SAS), versión 9.4. 

### Aspectos éticos

Para realizar la traducción y adaptación transcultural se solicitó autorización a la autora del instrumento original y se recibió retroalimentación positiva respecto a su uso. El proyecto fue aprobado por el Comité de Ética en Investigación, seguido de toda la información de aprobación (número CAAE: 68093623.2.0000.5374).

## Resultados

Las tres primeras etapas transcurrieron sin dificultades y fueron realizadas por profesionales experimentados en la materia.

Respecto a la etapa IV del estudio, alrededor de 16 expertos fueron invitados a participar en el Comité. De ellos, sólo 12 retornaron el contacto. Dos se negaron a participar y diez expertos aceptaron, sin embargo, sólo siete regresaron con el instrumento completo y compusieron el comité de expertos. Estaba formado exclusivamente por mujeres, dos trabajando en la región Nordeste de Brasil y el resto en la región Sudeste, tres postdoctoras, dos doctoras, una magíster y una especialista, con una media de 14,42 años (desviación estándar=3,35) de tiempo de formación en sus áreas. El Comité tenía un tiempo promedio de enseñanza de alrededor de 8 años (desviación estándar=5,44), 6,57 años (desviación estándar=3,40) trabajando con simulación clínica y con un total de 21 artículos publicados sobre el tema.

 El comité de expertos llevó a cabo dos rondas de análisis, una antes de la prueba previa final de la versión disponible y otra después de la prueba previa final. En la primera, se calcularon los valores IVC y Kappa modificado para todos los elementos del instrumento, como se muestra en la Tabla 1 . 

**Tabla 1:** - Resultados del Índice de Validez de Contenido (IVC ^*^ ) obtenidos en la primera ronda de evaluación de expertos. Campinas, SP, Brasil, 2023

	**Equivalencias**								**Claridad**		**Relevancia**	
**Ítem - Dominio**	**Semántica**		**Idiomática**		**Conceptual**		**Cultural**					
	**IVC** ^*^	***Kappa***	**IVC** ^*^	***Kappa***	**IVC** ^*^	***Kappa***	**IVC** ^*^	***Kappa***	**IVC** ^*^	***Kappa***	**IVC** ^*^	***Kappa***
**1.2 - Dominio 1**	0,857	0,857	0,893	0,893	0,893	0,893	0,929	0,928	0,893	0,893	1,00	1,00
**1.4 - Dominio 1**	0,857	0,857	0,893	0,893	0,929	0,928	0,929	0,929	0,964	0,964	0,857	0,857
**1.5 - Dominio 1**	0,893	0,893	0,929	0,929	0,964	0,964	0,964	0,964	0,929	0,929	0,964	0,964
**1.6 - Dominio 1**	0,857	0,857	0,929	0,929	1,00	1,00	1,00	1,00	1,00	1,00	1,00	1,00
**1.1 - Dominio 2**	0,893	0,893	0,964	0,964	0,964	0,964	0,964	0,964	0,964	0,964	0,964	0,964
**1.2 - Dominio 3**	0,893	0,893	0,929	0,929	0,964	0,964	0,964	0,964	0,964	0,964	0,964	0,964
**1.6 - Dominio 4**	0,893	0,893	0,929	0,929	0,964	0,964	0,964	0,964	0,929	0,929	1,00	1,00

* IVC = Índice de Validez de Contenido

 Once ítems (1.1, 1.3, 1.7 del Dominio 1; 1.3 del Dominio 2; 1.3, 1.5 del Dominio 3; 1.2, 1.4, 1.8 del Dominio 4; 1.1, 1.2 del Dominio 5) no aparecen en la Tabla 1 porque, a pesar de tener valores de IVC y de Kappa modificado aceptables, recibieron sugerencias gramaticales y fueron sometidos a reformulaciones específicas. 

 A pesar de no existir un IVC inferior a 0,9, el ítem que describe los conceptos de clasificación del facilitador recibió una sugerencia que fue aceptada como propuesta de modificación. La clasificación “principiante experimentado” en la versión síntesis fue sustituida por “principiante avanzado”, según lo sugerido por los expertos. En la Figura 2 se detallan todos los cambios realizados por el comité al evaluar la versión síntesis. 

 Para la realización de la prueba de la versión pre-final en la etapa V del estudio, se enviaron 134 invitaciones a expertos seleccionados por la Plataforma *Lattes* , y sólo 33 profesionales aceptaron participar. De estos participantes, el 66,7% (22) eran mujeres, y el 51,5% (17) tenía el doctorado como máximo nivel de formación. En cuanto a la graduación, el 63,6% (21) eran enfermeros, el 27,3% (9) eran médicos y el resto eran veterinario (1), fisioterapeuta (1) y pedagogo (1). La muestra de facilitadores de simulación tuvo un promedio de 19,5 años de formación en sus áreas (desviación estándar=12,91) y 14,2 años de tiempo docente (desviación estándar=12,11). Todos tenían un promedio de 6,7 años (desviación estándar = 4,08) de experiencia con simulación clínica. El tiempo promedio empleado en responder el instrumento fue de 11,6 minutos (desviación estándar=8,31). Todos los participantes consideraron que el instrumento era adecuado o parcialmente adecuado para evaluar la competencia del facilitador en simulación y encontraron los ítems comprensibles o parcialmente comprensibles. Se abrió un espacio para sugerencias, las que posteriormente fueron llevadas al comité de expertos en una segunda ronda de evaluación para análisis e incorporación de las propuestas realizadas. Se hicieron diez propuestas, pero el comité sólo aceptó dos. Ninguna de las propuestas afectó el contenido de los artículos. La Figura 3 muestra los cambios realizados en esta segunda ronda de evaluación del instrumento. 

**Figura 2:** - Versión síntesis y versión resultado de la evaluación del comité de expertos. Campinas, SP, Brasil, 2023

**Ítem**	**Versión síntesis**	**Versión resultante de la evaluación del comité de expertos**
Conceptos	Principiante (1) a principiante experimentado (2)	Principiante (1) a principiante avanzado (2)
1.1 Dominio 1 - Programación	- Puede identificar la necesidad de grupos pequeños junto a la cama. - Tiene creatividad en la planificación de actividades. - Puede programar la experiencia de aprendizaje de tal forma que sea óptima.	- Identifica la necesidad de grupos pequeños junto a la cama. - Demuestra creatividad en la planificación de actividades. - Programa la experiencia de aprendizaje de tal manera que sea óptima.
1.2 Dominio 1 - Objetivos de aprendizaje	- Contempla los dominios del aprendizaje cognitivo, afectivo y psicomotor. - Correlaciona los objetivos de todos los dominios con la educación o experiencia de los participantes.	- Aborda los dominios del aprendizaje cognitivo, afectivo y psicomotor. - Correlaciona los objetivos en todos los dominios con el nivel educativo o la experiencia de los participantes.
1.3 Dominio 1 - Proceso de planificación	- Informa al personal del laboratorio que se realizará una simulación. - Trabaja en conjunto con el equipo del laboratorio para alcanzar plenamente los objetivos de aprendizaje. - Analiza simulaciones clínicas previas, con el objetivo de mejorar la experiencia de aprendizaje.	- Informa al personal del laboratorio sobre los planes para realizar la simulación. - Colabora con el equipo del laboratorio para alcanzar plenamente los objetivos de aprendizaje. - Revisa simulaciones clínicas previas para mejorar la experiencia de aprendizaje.
1.4 Dominio 1 - Nivel de fidelidad (por ejemplo, ambiente, modalidad de simulación)	- Planifica un nivel de fidelidad que abarque los resultados deseados.	- Planifica el nivel de fidelidad que abarca los resultados deseados.
1.5 Dominio 1 - Disponibilidad de suministros/equipos	- Enumera los suministros y equipos necesarios en la simulación. - Organiza los materiales de aprendizaje necesarios por prioridad. - Desarrolla o mejora los materiales para estimular el pensamiento crítico en los alumnos.	- Enumera los suministros y equipos necesarios para la simulación. - Organiza los materiales didácticos por prioridad. - Desarrolla o mejora los materiales para estimular el pensamiento crítico en los participantes.
1.6 Dominio 1 - Requisitos de preparación	- Informa a los participantes de lo necesario para prepararse antes de la simulación. - Puede evaluar si los participantes están preparados para la simulación.	- Informa a los participantes sobre lo necesario para prepararse antes de la simulación. - Determina si los participantes están preparados para la simulación.
1.7 Dominio 1 - Métodos de evaluación	- Intenta utilizar buenas herramientas psicométricas de evaluación.	- Planea utilizar herramientas de evaluación con buenas propiedades psicométricas.
1.3 Dominio 2 - Identificación de roles	- Analiza qué rol se le debe dar a cada participante para optimizar el aprendizaje, considerando las fortalezas y debilidades ya identificadas.	- Analiza cuál rol se le debe dar a cada participante para optimizar el aprendizaje, considerando las fortalezas y debilidades ya identificadas.
1.3 Dominio 3 - Involucramiento de los participantes	- Durante la simulación, da buenas instrucciones o estímulos para que todos los participantes se involucren. - Emplea diferentes métodos para involucrar a aquellos que no participan mucho.	- Proporciona señales o estímulos para que todos los participantes se involucren durante la simulación. - Emplea diferentes métodos para involucrar a aquellos que no participan mucho en la simulación.
1.5 Dominio 3 - Tiempo/duración	- Continúa el escenario escrito, independientemente de la gestión del tiempo. - Se adapta a lo largo de la experiencia, buscando cubrir todos los objetivos de aprendizaje dentro del tiempo asignado.	- Continúa el escenario según el guión, sin preocuparse por la gestión del tiempo. - Se adapta a lo largo de la experiencia, buscando cubrir todos los objetivos de aprendizaje dentro del tiempo establecido.
1.2 Dominio 4 - Facilita la reflexión	- Estimula el análisis en profundidad de los procesos de elección y el pensamiento de orden superior.	- Estimula el análisis en profundidad de los procesos de toma de decisiones, del pensamiento crítico y de la resolución de problemas.
1.4 Dominio 4 - Escucha activa	- En la discusión aporta más que los propios participantes.	- Contribuye más que los propios participantes durante la discusión.
1.6 Dominio 4 - Objetivos de aprendizaje	- Presta atención a los eventos del escenario. - Puede evaluar si los objetivos de aprendizaje fueron asimilados. - Ayuda a los participantes a evaluar el nivel de asimilación de los objetivos de aprendizaje.	- Centra su atención en los eventos del escenario. - Puede evaluar si se lograron los objetivos de aprendizaje. - Ayuda a los participantes a determinar el logro de los objetivos de aprendizaje.
1.8 Dominio 4 - Resumen	- Ayuda a los participantes a resumir la simulación.	- Apoya a los participantes mientras resumen la simulación.
1.1 Dominio 5 - Experiencia	- Utiliza métodos diseñados para recopilar datos de los participantes, del personal y del cuerpo docente sobre la simulación.	- Utiliza métodos estructurados para recopilar datos de los participantes, del personal y del cuerpo docente sobre la simulación.
1.2 Dominio 5 - Participantes	- Utiliza métodos diseñados para recopilar datos sobre los participantes y el aprendizaje.	- Utiliza métodos estructurados para recopilar datos sobre los participantes y el aprendizaje.

**Figura 3:** - Versión pre-final y versión final resultante de la 2ª evaluación del comité de expertos. Campinas, SP, Brasil, 2023

**Ítem**	**Versión pre-final**	**Versión resultante de la 2da evaluación del comité de expertos (Versión final)**
Texto introductorio	Sin contenido	Estimado, si usted está realizando su autoevaluación o evaluando a un profesional utilizando la versión brasileña de la *Facilitator Competency Rubric* (FCR), informamos que las competencias de un facilitador se evalúan por medio de cinco dominios (preparación, *prebriefing* , facilitación, *debriefing* y evaluación). Para cada dominio, hay una lista de ítems. Recomendamos leer atentamente cada ítem y elegir la calificación que mejor le represente o represente al profesional que está evaluando: **(1) Principiante; (2) Principiante avanzado; (3) Competente; (4) Eficiente; (5) Experto** Al final, encontrarás la puntuación esperada para cada dominio.
Línea de puntuación en cada dominio	Plantilla para el total de las tres columnas — Sección “Preparación” 0-14 = “Principiante” a “Principiante avanzado” (necesita supervisión de un facilitador “Eficiente” a “Experto”). 15-27 = “Competente”. 28-35 = “Eficiente” a “Experto” (capaz de supervisar a un facilitador “Principiante” a “Principiante avanzado”).	Plantilla para el total de las tres columnas — Sección “Preparación” 0-14 = “Principiante” a “Principiante avanzado” (necesita supervisión de un facilitador “Eficiente” a “Experto”). 15-27 = “Competente”. 28-35 = “Eficiente” a “Experto” (capaz de supervisar a un facilitador “Principiante” a “Principiante avanzado”).

 En la Figura 3 se puede observar que se propuso la inclusión de un texto introductorio para guiar al facilitador en el llenado del instrumento, lo que logró un 100% de acuerdo entre los evaluadores. 

 Al final de todas las etapas, la versión final de la *Facilitator Competency Rubric* - Versión brasileña (FCR-VB) fue enviada a la autora del instrumento original, quien aprobó el proceso de adaptación. 

 En cuanto a la medición de confiabilidad de la FCR-VB, 52 facilitadores de simulación autocompletaron el instrumento. De estos participantes, el 78,85% (41) eran mujeres, el 73,1% (38) eran enfermeros, el 17,3% (9) eran médicos, el 5,77% (3) eran farmacéuticos, el 1,93% (1) eran nutricionistas y el 1,93% (1) eran biólogos. Alrededor del 38,5% (20) de estos profesionales tenían como máximo nivel de formación el Doctorado, el 25% (13) Especialización, el 23% (12) Maestría y el 13,5% (7) Postdoctorado. Además, los profesionales tenían un promedio de 17,19 años de tiempo de formación (desviación estándar=8,24), con un promedio de 10,98 años de experiencia en la docencia (desviación estándar=6,61), y habían utilizado la simulación en su práctica docente/laboral durante un tiempo promedio de 6,4 años (desviación estándar=5,28). La Tabla 2 presenta la clasificación de los facilitadores en simulación y los resultados de las medidas de confiabilidad de la versión brasileña de la FCR, verificadas con base en los datos alfa de Cronbach, medidos para cada dominio y para la escala general, y los valores del Coeficiente de Correlación Intraclase (ICC). 

**Tabla 2:** - Clasificación de los participantes en la prueba de las versiones pre-final y final y confiabilidad de la versión brasileña de la *Facilitator Competency Rubric* . Campinas, SP, Brasil, 2023

**Dominios de la escala**	**Clasificación**	**Prueba de versión pre-final (n=33)**	**Prueba de Confiabilidad (n=52)**	**Alfa de Cronbach (n=52)**	**ICC** ^*^ **(n=15)**
**Preparación**		Media ^†^ =24,54 (DE ^‡^ =7,30)	Media ^†^ =25,11 (DE ^‡^ =6,25)	0,911	0,95
	Principiante a Principiante Avanzado	12,1% (4)	3,8% (2)		
	Competente	51,5% (17)	57,7% (30)		
	Eficiente a Experto	36,4% (12)	38,5% (20)		
***Prebriefing***		Media ^†^ =14,69 ( ^‡^ DE=3,99)	Media ^†^ =13,82 (DE ^‡^ =4,26)	0,876	0,96
	Principiante a Principiante Avanzado	9,1% (3)	9,6% (5)		
	Competente	42,4% (14)	55,8% (29)		
	Eficiente a Experto	48,5% (16)	34,6% (18)		
**Facilitación**		Media ^†^ =22,42 (DE ^‡^ =5,89)	Media ^†^ =22,82 (DE ^‡^ =4,93)	0,906	0,95
	Principiante a Principiante Avanzado	12,1% (4)	1,9% (1)		
	Competente	27,3% (9)	53,8% (28)		
	Eficiente a Experto	60,6% (20)	44,3% (23)		
***Debriefing***		Media ^†^ =30,51 (DE ^‡^ =7,45)	Media ^†^ =29,56 (DE ^‡^ =7,08)	0,947	0,97
	Principiante a Principiante Avanzado	6,1% (2)	5,8% (3)		
	Competente	39,4% (13)	55,7% (29)		
	Eficiente a Experto	54,5% (18)	38,5% (20)		
**Evaluación**		Media ^†^ =14,06 (DE ^‡^ =3,85)	Media ^†^ =13,76 (DE ^‡^ =3,57)	0,878	0,97
	Principiante a Principiante Avanzado	9,1% (3)	9,6% (5)		
	Competente	48,5% (16)	61,5% (32)		
	Eficiente a Experto	42,4% (14)	28,9% (15)		
				Total=0,980	-

* ICC = Coeficiente de Correlación Intraclase;

† Media de la puntuación por dominio;

‡ DE = Desviación estándar por dominio

En la prueba de estabilidad de la escala, 35 de estos participantes aceptaron contribuir al estudio, sin embargo, sólo 15 regresaron con el instrumento completo después de 15 días de autoaplicación.

## Discusión

 La versión brasileña de la *Facilitator Competency Rubric* y su proceso de adaptación tuvieron éxito en diferentes áreas, además de presentar medidas confiables de consistencia interna y validez de contenido. 

 La adopción de un marco teórico y metodológico para el proceso de adaptación cultural de un instrumento está directamente asociada con la calidad del resultado obtenido luego de todas las etapas realizadas. El rigor y la complejidad necesarios para hacer que los ítems sean equivalentes entre las versiones original y adaptada de un instrumento son fundamentales para sustentar el uso calificado de este material. Garantizar la equivalencia entre las dos versiones comienza por elegir una metodología adecuada ^(^ ^7^ ^,^ ^15^ ^)^ . Todo este proceso fue seguido en la adaptación cultural de la FCR al contexto brasileño en este estudio. 

 Estudios que compararon métodos de adaptación cultural y sus validaciones demostraron que la etapa de *back translation* no es obligatoria, ya que no hay diferencia significativa en el resultado final del instrumento. Sin embargo, este paso puede ser útil como herramienta de comunicación con el autor del instrumento original ^(^ ^16^ ^-^ ^17^ ^)^ . Además, la participación efectiva del Comité de Expertos es fundamental para garantizar las equivalencias, y el retorno constante de las notas generadas en la etapa de prueba de la versión pre-final a este equipo multidisciplinario permite la exactitud del contenido de los ítems en la comparación entre las versiones original y traducida del instrumento ^(^ ^16^ ^-^ ^17^ ^)^ . En este estudio se cumplieron estas dos etapas, tal y como estaba previsto en el marco metodológico elegido ^(^ ^7^ ^)^ . 

 Cabe señalar que, para la composición del comité de expertos, se buscaron perfiles de participantes que se ajustaran a lo recomendado en la literatura ^(^ ^7^ ^-^ ^8^ ^)^ . Un grupo multidisciplinario que trabaja en diferentes frentes en estudios de simulación y validación refuerza la calidad y el cuidado que tuvo este estudio para garantizar una mejor validación del contenido del instrumento. Igualmente, elegir un valor de IVC de 0,9 aumenta la seguridad y el rigor del proceso, como se ha visto en otros estudios ^(^ ^18^ ^-^ ^20^ ^)^ . 

 La autora del instrumento original presentó en su estudio que la FCR fue diseñada para ser utilizada en la observación del facilitador de la simulación, lo que hace que el cuerpo docente sea más reacio a ser evaluado mientras desempeña su rol como profesor. Sin embargo, los resultados muestran que el uso autoaplicado del instrumento brinda más credibilidad a los hallazgos y orienta el desarrollo del cuerpo docente ^(^ ^5^ ^)^ . Por lo tanto, el presente estudio optó por la estrategia de la autoaplicación del instrumento para obtener los resultados. 

 Otra elección importante en el proceso de validación de la FCR-VB fue la inclusión de participantes con experiencia en simulación clínica y de un texto que orienta su realización. La elección de incluir sólo a personas con experiencia en el uso de la simulación se basó en el estudio alemán de traducción del instrumento, en el que los autores destacaron que la inclusión de participantes con poca o ninguna formación en simulación puede haber interferido con los resultados obtenidos ^(^ ^21^ ^)^ . Esta observación revela que el individuo que llena el instrumento necesita tener un mínimo de conocimiento sobre simulación clínica y sobre los dominios que el cuestionario pretende evaluar. 

Además, la inclusión del texto guía no interfirió directamente con el contenido del instrumento original, y también fue sugerida por los participantes de la investigación. Sin duda, la disponibilidad de una guía orientadora es fundamental para ayudar al profesional que aplica la estrategia docente a comprender los objetivos para autoevaluarse como facilitador.

 Los profesionales que participaron de la etapa V del estudio presentaron sugerencias relevantes respecto a la realidad del tema. Esto refuerza lo que la literatura comparte como importante: la inclusión del público objetivo en la recolección de datos y sus contribuciones para perfeccionar la comprensión del instrumento, y facilitar la adherencia a su uso en la práctica y obtener información y resultados veraces y de calidad ^(^ ^18^ ^-^ ^19^ ^)^ . 

 En el presente estudio, aunque no existen informes previos sobre el tiempo necesario para completar la FCR dentro o fuera del escenario de simulación ^(^ ^5^ ^,^ ^21^ ^)^ , hubo una gran disparidad en el tiempo registrado para completar el instrumento. Hubo una variación de 2 a 40 minutos en el tiempo para completar la escala, y los facilitadores realizaron comentarios sobre la gran cantidad de ítems (29) presentes en el instrumento, lo que puede estar asociado con la mayor cantidad de tiempo para terminar la autoevaluación. 

 En cuanto al análisis de consistencia interna, los resultados de Brasil son similares a los de la versión alemana ^(^ ^21^ ^)^ , con valores superiores a 0,8 para todos los dominios. No fue posible comparar con los valores encontrados en el estudio original, ya que la medición de la consistencia interna se realizó de manera diferente, utilizando el Coeficiente G, que mostró resultados de bueno a excelente ^(^ ^5^ ^)^ , también verificados en la versión brasileña. 

 Para medir la estabilidad de la escala, verificada mediante prueba-reprueba, las tres versiones presentaron ICC por encima de los valores de referencia de 0,70 ^(^ ^14^ ^-^ ^15^ ^)^ , lo que resalta cuán estables son las medidas que el instrumento evalúa en momentos distintos. 

 Este estudio no estuvo exento de limitaciones. En la etapa de prueba de la versión pre-final no fue posible calcular la tasa de respuesta, ya que se adoptó el reclutamiento en bola de nieve para el autocompletado del instrumento, lo que puede considerarse un sesgo de muestreo. Asimismo, el número de individuos que participaron en la etapa de prueba-reprueba para medir la confiabilidad fue inferior al recomendado en la literatura, que prevé un mínimo de 50 individuos ^(^ ^8^ ^,^ ^13^ ^-^ ^14^ ^)^ . Recomendamos que, en futuros estudios y con un tamaño de muestra mayor, se realicen evaluaciones de la estructura de la escala, mediante análisis factorial exploratorio, seguido de análisis factorial confirmatorio, para confirmar la disposición de los ítems en los dominios del instrumento. 

Proporcionar a la versión brasileña de la FCR las propiedades de medidas probadas y validadas para el escenario académico e institucional contribuye al avance de buenas prácticas de simulación clínica en ámbitos de práctica cada vez más utilizados en el área de la salud. Utilizando la rúbrica, el facilitador podrá reconocer sus fortalezas y limitaciones al aplicar la metodología de simulación, es decir, la versión brasileña de la FCR puede ser utilizada en el desarrollo de profesionales como facilitadores en simulación clínica, puntuando las habilidades que deben aún ser desarrolladas. Con ello, los profesionales que pretendan actuar en el rol de facilitadores de manera efectiva y eficiente podrán buscar una capacitación más relevante a sus objetivos para promover mejores condiciones de enseñanza y aprendizaje, utilizando todo el potencial que ofrece la simulación clínica. Tampoco podemos excluir las ventajas de hacer avanzar la ciencia en el campo de la investigación de simulación con el uso de este instrumento.

## Conclusión

El proceso de traducción y adaptación de la FCR siguió cuidadosamente los pasos descritos como referencia en la literatura internacional. La inclusión de este instrumento en el escenario nacional de simulación clínica brasileña puede aumentar el conocimiento sobre las competencias de facilitadores y el desarrollo de nuevas habilidades para los profesionales que utilizan esta estrategia de enseñanza. La versión brasileña de la FCR demostró ser adecuada para su uso y autoevaluación. Se recomiendan estudios con un tamaño de muestra mayor y que evalúen diferentes propiedades de medición para aumentar la precisión y validez del instrumento.

# Translation: Adaptação cultural e avaliação das propriedades de medida da ***Facilitator Competency Rubric*** para facilitadores de simulação clínica

**DOI:** 10.1590/1518-8345.7214.4258

**Authors:** Daniel Gonçalves Campos, Juliany Lino Gomes Silva, Ruana Luiz Ferreira da Silva, Angélica Olivetto Almeida, Ana Luísa Brandão de Carvalho Lira, Ana Railka de Souza Oliveira-Kumakura

**Affiliations:** 1 Universidade Estadual de Campinas, Faculdade de Enfermagem, Campinas, SP, Brasil.; 2 Universidade Federal do Ceará, Departamento de Enfermagem, Fortaleza, CE, Brasil.; 3 Université Paris Cité, Département Universitaire en Sciences Infirmières, Paris, França.

**Keywords:** Treinamento por Simulação, Inquéritos e Questionários, Educação, Ensino, Docentes, Competência Profissional

## Abstract

**Objetivo:**

traduzir e adaptar a *Facilitator Competency Rubric* para o idioma português e a cultura brasileira, e analisar as propriedades de medida.

**Método:**

estudo metodológico que cumpriu as etapas de tradução, síntese das traduções, *back translation* , revisão por um comitê de especialistas composto por sete profissionais, teste da versão pré-final com 33 facilitadores de simulação e submissão à autora do instrumento original. Para a validação de conteúdo, foram calculados o Índice de Validade de Conteúdo e o Coeficiente de Kappa modificado. Para a confiabilidade foram avaliados o α de Cronbach e o Coeficiente de Correlação Intraclasse por 52 e 15 facilitadores de simulação, respectivamente.

**Resultados:**

foram realizadas duas rodadas de avaliação do conteúdo, com mudança de 19 itens na primeira avaliação e três itens na segunda. A escala geral alcançou α de Cronbach de 0,98 e Coeficientes de Correlação Intraclasse de 0,95 a 0,97.

**Conclusão:**

a *Facilitator Competency Rubric* foi traduzida e adaptada culturalmente à realidade brasileira e apresentou validade de conteúdo, confiabilidade e estabilidade, com resultados seguros para uso no ensino e na pesquisa.

## Destaques

**(1)** A versão brasileira da FCR tem seu conteúdo válido e é confiável para uso nacional.

**(2)** A FCR adaptada ao contexto brasileiro mede a competência do facilitador de simulação.

**(3)** Medir a competência com a FCR favorece o cenário de capacitação docente em simulação.

## Introdução

 A aprendizagem baseada em simulação teve um advento nos últimos anos e tem sido uma estratégia de ensino bastante utilizada por programas de graduação de diversas áreas da saúde ^(^ ^1^ ^-^ ^2^ ^)^ . Pesquisadores apontam que cerca de metade das horas referentes à experiência clínica tradicional podem ser substituídas por experiências clínicas simuladas se certas condições de boa execução forem atendidas, como treinamento formal na pedagogia de simulação, número adequado de professores para auxiliar os estudantes, presença de especialistas na condução de um *debriefing* baseado em evidências e recursos materiais adequados para tornar o ambiente o mais realista possível ^(^ ^2^ ^)^ . 

 Para tanto, diferentes instrumentos de medidas têm sido desenvolvidos ao longo dos anos para avaliar elementos que são necessários para a implementação dessa estratégia de ensino ou para avaliar os seus resultados, principalmente dos participantes ^(^ ^3^ ^)^ . Por exemplo, a *National League for Nursing* (NLN) e a *International Nursing Association for Clinical and Simulation Learning* (INACSL) disponibilizam instrumentos que foram traduzidos e validados para diferentes culturas e que focam na avaliação do desempenho de habilidades, satisfação, percepção sobre a experiência educacional, conhecimento/aprendizagem, pensamento crítico/julgamento clínico, autoconfiança/autoeficácia, *debriefing* , competência do facilitador, e na avaliação do nível de organização quanto ao *design* do cenário simulado ^(^ ^4^ ^)^ . 

 A maioria desses instrumentos avalia a experiência do estudante com a simulação, no entanto, a avaliação do indivíduo que facilita a simulação ainda é indefinida, apesar das evidências de seu desempenho estarem inseridas em outros tipos de ferramentas ^(^ ^5^ ^)^ . Entre as escalas com foco no facilitador, encontra-se a *Facilitator Competency Rubric* (FCR). Ela foi desenvolvida para avaliar e medir o nível de competência do educador ao facilitar o aprendizado com o uso da simulação, e apresenta versões nos idiomas inglês e alemão. O referencial teórico de Benner é utilizado para diferenciar os níveis de competência do facilitador, sendo representados por: iniciante, iniciante avançado, competente, proficiente e especialista ^(^ ^5^ ^)^ . 

 O facilitador é considerado o profissional educador que tem como responsabilidade acompanhar e garantir toda a experiência baseada em simulação da maneira mais segura possível para que os aprendizes trabalhem de forma coesa, com foco na compreensão dos objetivos de aprendizagem e desenvolvimento de um plano para o alcance dos resultados desejados. Ser um facilitador requer formação e habilidades para condução, apoio e busca de maneiras de ajudar os aprendizes a alcançarem os resultados esperados ^(^ ^6^ ^)^ . 

 O facilitador iniciante é uma pessoa sem experiência cujos comportamentos e decisões são direcionados por regras preestabelecidas e que pode aprender as tarefas, mas não sabe o que fazer com as informações obtidas. Já o iniciante avançado apresenta um desempenho aceitável. O competente consegue desenvolver, implementar e priorizar uma estratégia de ação para a resolução de um problema, sentindo-se no domínio da situação. O facilitador proficiente consegue ver a situação numa perspectiva mais global frente à sua experiência, agindo de maneira mais rápida e flexível em comparação com o competente. Por último, o especialista não se sustenta em regras, mas em sua experiência e intuição. Dentro do cenário de educação e ensino, os profissionais podem percorrer esses níveis à medida que mudam de responsabilidade no trabalho ou aprendem novas estratégias de ensino ^(^ ^5^ ^)^ . 

 O instrumento FCR é composto por cinco dimensões: preparo, *prebriefing* , facilitação, *debriefing* e avaliação. Para essas dimensões, tem-se um total de 29 itens, que podem ser classificados numa escala ordinal de iniciante (1) a especialista (5). O estudo de desenvolvimento da escala verificou que a confiabilidade entre avaliadores foi boa. Os coeficientes de Generalização (G), usados para avaliar a confiabilidade entre avaliadores e para determinar a quantidade de variância atribuível a eles, variaram de bom a excelente (0,80 a 0,99), e os itens prazo, dia da semana, hora do dia e tipos de simulação foram preditores significativos da pontuação global do instrumento ^(^ ^5^ ^)^ . 

 Na perspectiva de que a FCR é um instrumento que, no cenário brasileiro, pode fornecer orientação para a capacitação do corpo docente e ser utilizado como ferramenta de autoavaliação ou de avaliação formativa ou somativa de um facilitador de simulação ^(^ ^5^ ^)^ , propõe-se a realização deste estudo. O objetivo foi traduzir e adaptar a *Facilitator Competency Rubric* para o idioma português e a cultura brasileira, e analisar as propriedades de medida. 

## Método

### Delineamento do estudo

 Estudo metodológico, que cumpriu as seguintes etapas: tradução, síntese das traduções, *back translation* , revisão por um comitê de especialistas, teste da versão final e submissão à autora do instrumento original ^(^ ^7^ ^)^ . Além disso, foram realizadas a validação de conteúdo e a medida de confiabilidade. A pesquisa foi descrita a partir dos critérios adotados pelo *checklist* do *COnsensus-Based Standards for the selection of health Measurement INstruments* (COSMIN) ^(^ ^8^ ^)^ . A Figura 1 elucida o fluxograma metodológico do estudo. 

**Figura 1:** - Fluxograma metodológico do estudo. Campinas, SP, Brasil, 2023

### Período

O período de coleta de dados se deu após a aprovação do estudo em Comitê de Ética em Pesquisa e ocorreu entre os meses de abril e setembro de 2023.

### Etapa I – Traduções

 Foi realizada a tradução do instrumento do inglês para português brasileiro, obtendo-se duas versões iniciais: uma delas feita por um tradutor com conhecimento das terminologias de simulação e área da saúde e outra realizada por um tradutor sem conhecimento na área. Ambos profissionais são fluentes no idioma original do instrumento e de naturalidade brasileira, com vivência na língua-alvo da tradução, o português brasileiro. Portanto, foi de interesse a identificação de possíveis diferenças entre palavras e frases do instrumento, seguindo o modelo proposto ^(^ ^7^ ^)^ . Surgiram, assim, duas versões, a saber: instrumento 1 (INST1) e instrumento 2 (INST2). A primeira com uma tradução mais literal do instrumento e a segunda com uma tradução mais interpretativa, condizente com a linguagem da população. 

### Etapa II – Síntese das traduções

As versões INST1 e INST2 foram comparadas com o instrumento original e sintetizadas, gerando uma versão única, chamada instrumento 12 (INST12). Essa etapa foi realizada por um terceiro tradutor, que possuía o português como língua materna e fluência em inglês.

### Etapa III – *Back translation*

 A versão INST12 obtida pela síntese foi traduzida para a língua original da escala, gerando duas versões: *back translation* 1 (BINST1) e *back translation* 2 (BINST2). Essa etapa foi realizada por dois tradutores bilíngues cuja língua materna era a mesma do instrumento original (inglês). Ambos tradutores não apresentavam conhecimento prévio sobre o instrumento e sua utilização, e não possuíam formação na área. Com este processo, a intenção foi observar imprecisões conceituais na tradução e garantir que a versão traduzida fosse clara e precisa quanto ao conteúdo da versão original do instrumento. 

### Etapa IV – Revisão e validação de conteúdo por um comitê de especialistas

 De acordo com o referencial de tradução utilizado ^(^ ^7^ ^)^ , esta fase é essencial para o alcance da tradução transcultural do instrumento. A formação de um comitê de especialistas é necessária para avaliar todas as versões obtidas através das etapas anteriores. 

 É recomendado que o número de participantes especialistas em um comitê seja de entre cinco e dez ^(^ ^7^ ^-^ ^9^ ^)^ . Os membros desse comitê foram convidados a participar a partir da análise do currículo na Plataforma *Lattes* . Como critério de inclusão para o convite dos especialistas foram utilizados os seguintes filtros: “Simulação Clínica” e “Estudos de Validação”. Foram extraídos aqueles currículos que estavam atualizados no ano de 2023. De maneira intencional, foram selecionados os especialistas para a composição do comitê com, no mínimo, três anos de experiência na área de simulação clínica ou em estudos de validação, experiência na prática clínica, pesquisas ou publicações sobre o tema e perícia na estrutura conceitual em simulação. 

 O comitê foi composto por especialistas com experiência em simulação clínica, na elaboração de cenários e em ensino em saúde, que aceitaram o convite. O objetivo foi avaliar todas as versões (INST1, INST2, INST12, BINST1 e BINST2), comparando-as com o instrumento original, para se alcançar a equivalência semântica, idiomática, experiencial e conceitual entre a versão original do instrumento e a da língua portuguesa ^(^ ^7^ ^)^ . 

 A equivalência semântica determina se há paridade no significado das palavras e na representação gramatical. Na equivalência idiomática há uma avaliação de expressões da língua original do instrumento, comparando-se com as adaptadas do instrumento traduzido para língua portuguesa. Já na equivalência experiencial é avaliado se o conteúdo descrito no instrumento é condizente para utilização na população-alvo. Por fim, na equivalência conceitual foi observado se algumas palavras possuem significados semelhantes ou se possuem a mesma importância nas línguas e culturas diferentes ^(^ ^7^ ^)^ . 

 Em seguida, esse mesmo grupo de especialistas realizou a validação do conteúdo da escala. Foram consideradas as seguintes propriedades da psicometria: abrangência da escala, clareza e relevância (ou pertinência) de cada item. Na avaliação da abrangência do instrumento, objetivou-se compreender se cada domínio ou conceito foi adequadamente atingido pelo conjunto de itens apresentados. Com relação à clareza, o foco foi na compreensão e redação dos itens, com o intuito de avaliar se cada um deles expressou efetivamente aquilo que se esperava medir. Já na relevância ou pertinência, avaliou-se a importância dos itens para o alcance dos objetivos propostos com a aplicação da escala, e se refletiam os conceitos envolvidos ^(^ ^7^ ^-^ ^8^ ^,^ ^10^ ^)^ . 

Os especialistas preencheram um documento para medir a proporção de concordância entre eles sobre os aspectos do instrumento e seus itens, por meio de uma escala ordinal com pontuação de 1 a 4, sendo 1 = não relevante/claro/equivalente, 2 = item necessita de grande revisão para ser relevante/claro/equivalente, 3 = item necessita de pequena revisão para ser relevante/claro/equivalente e 4 = item relevante/claro/equivalente. Em seguida, foi solicitada a avaliação da abrangência da escala, que utilizou uma escala ordinal de quatro pontos, sendo 1 = não abrangente, 2 = escala necessita de grande revisão para ser abrangente, 3 = escala necessita de pequena revisão para ser abrangente e 4 = escala abrangente.

 Apontamentos pertinentes acerca dos itens foram avaliados pelo pesquisador principal do estudo, que compilou as sugestões e enviou por *e-mail* ao Comitê de Especialistas para nova apreciação de forma *online* da versão traduzida. 

 Portanto, ao término desta etapa, foi disponibilizada a versão pré-final chamada *Facilitator Competency Rubric – Brazilian Version* (FCR-BV), que se consolidou pelo consenso de ideias e opiniões dos especialistas. 

### Etapa V – Teste da versão pré-final

 O teste da versão pré-final foi importante para a estabilização do instrumento, e é uma etapa em que o mesmo é exposto a novas avaliações ^(^ ^7^ ^)^ . 

 Para a realização do teste foram convidados profissionais da saúde que fossem facilitadores de simulação clínica, selecionados a partir da Plataforma *Lattes* , utilizando como critério de inclusão as palavras “Simulação Clínica”. Além disso, foram contatados facilitadores de simulação membros da Sociedade Brasileira de Simulação na Saúde (SOBRASSIM). Para ambos foi solicitada a indicação de indivíduos que pudessem participar desta etapa do estudo, por meio do método de recrutamento em bola de neve ^(^ ^11^ ^)^ . 

 É recomendado que, para esse processo, o instrumento seja aplicado idealmente em uma população de 30 a 40 indivíduos ^(^ ^7^ ^-^ ^8^ ^)^ . Foram então selecionados, de maneira aleatória, no mínimo 30 profissionais para a autoaplicação do instrumento na versão pré-final. Os profissionais que aceitaram o convite para participação nesta etapa receberam a versão pré-final da *Facilitator Competency Rubric -* *Brazilian Version* para auto aplicação; uma ficha de caracterização sobre o seu perfil profissional, que incluiu tempo de experiência em simulação, características sociodemográficas e de ensino; e um documento no qual pudessem compartilhar sobre a clareza, a compreensão e a facilidade do instrumento, tempo de preenchimento e um espaço para sugestões e mudanças. No caso de sugestões que pudessem alterar o conteúdo do item, o mesmo foi novamente encaminhado para a revisão do comitê de especialistas para alinhamento e possível adequação ^(^ ^8^ ^)^ . 

### Etapa VI – Submissão e validação do instrumento pelos autores

Nesta fase, foi enviado um relatório para a autora do instrumento original, contendo todo o processo de formação da versão final, incluindo as versões anteriores originadas das traduções diversas.

### Etapa de validação - Teste de confiabilidade

 Foi avaliada a confiabilidade por meio da análise da consistência interna e da estabilidade da rubrica. A abordagem desses indivíduos ocorreu de duas formas: em um evento internacional sobre simulação realística e pela seleção do currículo *Lattes* , conforme descrito na etapa V. Os participantes foram convidados a preencher um formulário na plataforma *Google Forms* , com o mesmo instrumento de caracterização utilizado na fase de pré-teste e versão final do instrumento FCR-VB. Os participantes que concordaram foram contatados novamente depois de 15 dias para uma nova aplicação da escala para avaliação do teste-reteste ^(^ ^10^ ^,^ ^12^ ^-^ ^13^ ^)^ . 

### Análise dos resultados e estatística

 As equivalências e a validação de conteúdo foram avaliadas de maneira quantitativa por meio do cálculo do Índice de Validade de Conteúdo (IVC) e do Coeficiente Kappa modificado. Foram considerados como aceitáveis valores mínimos de 0,90 e 0,74, respectivamente ^(^ ^8^ ^,^ ^13^ ^)^ . Para os itens que não alcançaram o escore mínimo estabelecido, as sugestões dos especialistas foram analisadas e incorporadas ao instrumento ^(^ ^8^ ^,^ ^14^ ^)^ . 

 Os dados obtidos foram tabulados em planilhas do *Microsoft Excel for Windows* ^®^ , nas quais as medidas das variáveis quantitativas e as medidas de frequência das variáveis qualitativas foram calculadas. O IVC foi calculado pela soma das concordâncias dos itens classificados como “3” ou “4” pelos especialistas e dividido pelo número total de respostas do instrumento. Aqueles itens que receberam a pontuação “1” ou “2” foram revisados pelo Comitê. Já o Coeficiente Kappa modificado foi obtido pela razão da proporção de vezes que os especialistas concordaram com a proporção máxima de vezes que eles poderiam concordar ^(^ ^8^ ^,^ ^10^ ^,^ ^13^ ^)^ . 

 A medida de confiabilidade da escala foi realizada pela análise da consistência interna, através do Coeficiente alfa de Cronbach ^(^ ^10^ ^)^ . Considerou-se os valores superiores a 0,7 para a avaliação da consistência do instrumento ^(^ ^10^ ^,^ ^13^ ^)^ . 

 Para avaliar a concordância entre as medidas obtidas no teste e reteste com relação aos escores do instrumento foi aplicado o Coeficiente de Correlação Intraclasse ^(^ ^10^ ^,^ ^13^ ^)^ . A escala foi aplicada em dois momentos distintos, com o intuito de verificar se os resultados obtidos seriam similares, ou seja, estimar se havia consistência frente às repetições das medidas. É esperado que a competência do facilitador seja a mesma nos dois momentos da aplicação do instrumento ^(^ ^10^ ^,^ ^13^ ^)^ . Valores maiores ou iguais a 0,70 foram considerados como indicativos de boa confiabilidade ^(^ ^8^ ^,^ ^10^ ^,^ ^13^ ^)^ . 

 Em todas as análises foi utilizado o *Statistical Analysis Software* ^®^ (SAS), versão 9.4. 

### Aspectos éticos

Para a realização da tradução e adaptação transcultural foi solicitada a autorização da autora do instrumento original e recebida a devolutiva positiva sobre o uso. O projeto foi aprovado pelo Comitê de Ética em Pesquisa, seguido de todas as informações de aprovação (número CAAE: 68093623.2.0000.5374).

## Resultados

As três primeiras etapas transcorreram sem dificuldade e foram realizadas por profissionais experientes na área.

Referente à etapa IV do estudo, cerca de 16 especialistas foram convidados a participar do comitê. Desses, apenas 12 retornaram o contato. Dois se negaram a participar, e dez especialistas aceitaram, no entanto, apenas sete retornaram com o instrumento preenchido e compuseram o comitê de especialistas. Este foi formado exclusivamente por mulheres, sendo duas atuantes na região Nordeste do Brasil e o restante na região Sudeste, três pós-doutoras, duas doutoras, uma mestra e uma especialista, com média de 14,42 anos (desvio-padrão=3,35) de tempo de formação em suas áreas. O comitê tinha média de oito (desvio-padrão=5,44) anos de tempo de docência; com média de 6,57 (desvio-padrão=3,40) anos trabalhando com simulação clínica e com um total de 21 artigos publicados sobre o tema.

 Foram realizadas duas rodadas de análise pelo comitê de especialistas, uma antes do pré-teste final da versão disponibilizada e outra depois do pré-teste final. Na primeira, foram calculados os valores de IVC e de Kappa modificado para todos os itens do instrumento, como mostra a Tabela 1 . 

**Tabela 1:** - Resultados do Índice de Validade de Conteúdo (IVC ^*^ ) obtidos na primeira rodada de avaliação de especialistas. Campinas, SP, Brasil, 2023

	**Equivalências**								**Clareza**		**Relevância**	
**Item - Domínio**	**Semântica**		**Idiomática**		**Conceitual**		**Cultural**					
	**IVC** ^*^	Kappa	**IVC** ^*^	Kappa	**IVC** ^*^	Kappa	**IVC** ^*^	Kappa	**IVC** ^*^	Kappa	**IVC** ^*^	Kappa
**1.2 - Domínio 1**	0,857	0,857	0,893	0,893	0,893	0,893	0,929	0,928	0,893	0,893	1,00	1,00
**1.4 - Domínio 1**	0,857	0,857	0,893	0,893	0,929	0,928	0,929	0,929	0,964	0,964	0,857	0,857
**1.5 - Domínio 1**	0,893	0,893	0,929	0,929	0,964	0,964	0,964	0,964	0,929	0,929	0,964	0,964
**1.6 - Domínio 1**	0,857	0,857	0,929	0,929	1,00	1,00	1,00	1,00	1,00	1,00	1,00	1,00
**1.1 - Domínio 2**	0,893	0,893	0,964	0,964	0,964	0,964	0,964	0,964	0,964	0,964	0,964	0,964
**1.2 - Domínio 3**	0,893	0,893	0,929	0,929	0,964	0,964	0,964	0,964	0,964	0,964	0,964	0,964
**1.6 - Domínio 4**	0,893	0,893	0,929	0,929	0,964	0,964	0,964	0,964	0,929	0,929	1,00	1,00

* IVC = Índice de Validade de Conteúdo

 Onze itens (1.1, 1.3, 1.7 do Domínio 1; 1.3 do Domínio 2; 1.3, 1.5 do Domínio 3; 1.2, 1.4, 1.8 do Domínio 4; 1.1, 1.2 do Domínio 5) não aparecem na Tabela 1 pois, apesar de terem tido valores de IVC e Kappa modificado aceitáveis, receberam sugestões gramaticais e sofreram reformulações pontuais. 

 Apesar de não haver IVC inferior a 0,9, o item que descreve os conceitos de classificação do facilitador recebeu uma sugestão que foi aceita como proposta de modificação. A classificação “iniciante experiente” na versão síntese foi substituída por “iniciante avançado”, conforme sugestão dos especialistas. Na Figura 2 são detalhadas todas as modificações realizadas pelo comitê ao avaliar a versão síntese. 

 Para a realização do teste da versão pré-final na etapa V do estudo, foram enviados 134 convites para especialistas selecionados pela Plataforma *Lattes* , e apenas 33 profissionais aceitaram participar. Desses participantes, 66,7% (22) eram do sexo feminino, e 51,5% (17) tinham o doutorado como nível mais elevado de formação. Com relação à graduação, 63,6% (21) eram enfermeiros, 27,3% (9) eram médicos e o restante era médico veterinário (1), fisioterapeuta (1) e pedagogo (1). A amostra de facilitadores em simulação tinha média de 19,5 anos de formação em suas áreas (desvio-padrão=12,91) e 14,2 anos de tempo em docência (desvio-padrão=12,11). Todos tinham uma média de 6,7 anos (desvio-padrão=4,08) de experiência com simulação clínica. A média de tempo gasto para responder o instrumento foi de 11,6 minutos (desvio-padrão=8,31). Todos os participantes consideraram o instrumento adequado ou parcialmente adequado para avaliar a competência do facilitador em simulação e acharam os itens compreensíveis ou parcialmente compreensíveis. Foi aberto um espaço para sugestões, que foram posteriormente levadas ao comitê de especialistas em uma segunda rodada de avaliação para análise e incorporação de propostas feitas. Foram realizadas 10 propostas, no entanto, apenas duas foram acatadas pelo comitê. Nenhuma das propostas afetou o conteúdo dos itens. A Figura 3 mostra as mudanças realizadas nessa segunda rodada de avaliação do instrumento. 

**Figura 2:** - Versão síntese e versão resultado da avaliação do comitê de especialistas. Campinas, SP, Brasil, 2023

**Item**	**Versão síntese**	**Versão resultante da avaliação do comitê de especialistas**
Conceitos	Iniciante (1) a iniciante experiente (2)	Iniciante (1) a iniciante avançado (2)
1.1 Domínio 1 - Programação	- Consegue identificar a necessidade de pequenos grupos à beira do leito. - Tem criatividade para programar as atividades. - Consegue programar a experiência de aprendizagem de tal modo que seja ideal.	- Identifica a necessidade de pequenos grupos à beira do leito. - Demonstra criatividade para programar as atividades. - Programa a experiência de aprendizagem de tal modo que seja ideal.
1.2 Domínio 1 - Objetivos de aprendizagem	- Contempla os domínios de aprendizagem cognitivo, afetivo e psicomotor. - Correlaciona os objetivos de todos os domínios à escolaridade ou à experiência dos participantes.	- Aborda os domínios de aprendizagem cognitivo, afetivo e psicomotor. - Correlaciona os objetivos de todos os domínios ao nível educacional
1.3 Domínio 1 - Processo de planejamento	- Informa a equipe do laboratório de que será conduzida uma simulação. - Trabalha junto com a equipe do laboratório pelo pleno cumprimento dos objetivos de aprendizagem. - Analisa as simulações clínicas prévias, visando melhorar a experiência de aprendizagem.	- Informa a equipe do laboratório sobre os planos para conduzir a simulação. - Colabora com a equipe do laboratório para o pleno cumprimento dos objetivos de aprendizagem. - Revisa as simulações clínicas prévias para melhorar a experiência de aprendizagem.
1.4 Domínio 1 - Nível de fidelidade (p. ex., ambiente, modalidade de simulação)	- Planeja um nível de fidelidade que contemple os desfechos desejados.	- Planeja o nível de fidelidade que contemple os resultados desejados.
1.5 Domínio 1 - Disponibilidade de suprimentos/equipamentos	- Elenca os suprimentos e equipamentos necessários na simulação. - Organiza os materiais de aprendizagem necessários por prioridade. - Desenvolve ou melhora os materiais para estimular o pensamento crítico dos aprendizes.	- Elenca os suprimentos e equipamentos necessários para a simulação. - Organiza por prioridade os materiais de ensino. - Desenvolve ou melhora os materiais para estimular o pensamento crítico dos participantes.
1.6 Domínio 1 - Requisitos de preparo	- Informa os participantes do necessário para que se preparem antes da simulação. - Consegue avaliar se os participantes estão preparados para a simulação.	- Informa os participantes sobre o necessário para que se preparem antes da simulação. - Determina se os participantes estão preparados para a simulação.
1.7 Domínio 1 - Métodos de avaliação	- Busca usar boas ferramentas psicométricas de avaliação.	- Planeja usar ferramentas de avaliação com boas propriedades psicométricas.
1.3 Domínio 2 - Identificação de papéis	- Analisa que papel deve ser dado a qual participante para otimizar a aprendizagem, considerando os pontos fortes e fracos já identificados.	- Analisa qual papel deve ser dado a cada participante para otimizar a aprendizagem, considerando os pontos fortes e fracos já identificados.
1.3 Domínio 3 - Envolvimento dos participantes	- Durante a simulação, dá boas indicações ou estímulos para que todos os participantes se envolvam. - Emprega métodos diversos para envolver aqueles que não estão participando muito.	- Fornece pistas ou estímulos para que todos os participantes se envolvam durante a simulação. - Emprega métodos diversos para envolver aqueles que não estão participando muito da simulação.
1.5 Domínio 3 - Tempo/duração	- Dá continuidade no cenário escrito, independentemente do manejo de tempo. - Adapta-se no decorrer da experiência, buscando contemplar todos os objetivos de aprendizagem dentro do tempo alocado.	- Dá continuidade no cenário conforme o *script* , sem se preocupar com o gerenciamento do tempo. - Adapta-se no decorrer da experiência, buscando contemplar todos os objetivos de aprendizagem dentro do tempo estabelecido.
1.2 Domínio 4 - Facilita a reflexão	- Estimula a análise profunda dos processos de escolha e o pensamento de ordem superior.	- Estimula a análise profunda dos processos de tomada de decisão, do pensamento crítico e da resolução de problemas.
1.4 Domínio 4 - Escuta ativa	- Na discussão, contribui mais que os próprios participantes.	- Contribui mais que os próprios participantes durante a discussão.
1.6 Domínio 4 - Objetivos de aprendizagem	- Atenção nos eventos do cenário. - Consegue avaliar se os objetivos de aprendizagem foram assimilados. - Auxilia os participantes na avaliação do nível de assimilação dos objetivos de aprendizagem.	- Centra a sua atenção nos eventos do cenário. - Consegue avaliar se os objetivos de aprendizagem foram alcançados. - Auxilia os participantes a determinar o alcance dos objetivos de aprendizagem.
1.8 Domínio 4 - Resumo	- Ajuda os participantes para que eles façam um resumo da simulação.	- Apoia os participantes enquanto eles resumem a simulação.
1.1 Domínio 5 - Experiência	- Usa métodos desenhados para coletar dados dos participantes, da equipe e do corpo docente sobre a simulação.	- Usa métodos estruturados para coletar dados dos participantes, da equipe e do corpo docente sobre a simulação.
1.2 Domínio 5 - Participantes	- Usa métodos desenhados para coletar dados sobre os participantes e a aprendizagem.	- Usa métodos estruturados para coletar dados sobre os participantes e a aprendizagem.

**Figura 3:** - Versão pré-final e versão final resultada da 2ª avaliação do comitê de especialistas. Campinas, SP, Brasil, 2023

**Item**	**Versão pré-final**	**Versão resultante da 2ª avaliação do comitê de especialistas (Versão final)**
Texto introdutório	Nenhum conteúdo	Prezado, caso você esteja realizando sua autoavaliação ou avaliando um profissional por meio da versão brasileira da *Facilitator Competency Rubric* (FCR), informamos que as competências para um facilitador são avaliadas por meio de cinco domínios (preparo, *prebriefing* , facilitação, *debriefing* e avaliação). Para cada domínio, há uma lista de itens. Recomendamos que você leia atentamente cada item e escolha a classificação que melhor o representa ou representa o profissional que você está avaliando: **(1) Iniciante; (2) Iniciante Avançado; (3) Competente; (4) Proficiente; (5) Especialista** Ao final, você encontrará a pontuação esperada para cada domínio.
Linha da pontuação em cada domínio	Gabarito do total das três colunas — Seção “Preparo” 0-14 = “Iniciante” a “Iniciante avançado” (carece de supervisão de um facilitador “Proficiente” a “Especialista”). 15-27 = “Competente”. 28-35 = “Proficiente” a “Especialista” (apto a supervisionar um facilitador “Iniciante” a “Iniciante avançado”).	Gabarito do total das três colunas — Seção “Preparo” 0-14 = “Iniciante” a “Iniciante avançado” (necessita de supervisão de um facilitador “Proficiente” a “Especialista”). 15-27 = “Competente”. 28-35 = “Proficiente” a “Especialista” (apto a supervisionar um facilitador “Iniciante” a “Iniciante avançado”).

 Na Figura 3 , observa-se que foi proposta a inclusão de um texto introdutório para orientar o facilitador sobre o preenchimento do instrumento, o que obteve 100% de concordância entre os avaliadores. 

 Ao término de todas as etapas, a versão final da *Facilitator Competency Rubric* - Versão brasileira (FCR-VB) foi encaminhada para a autora do instrumento original, que aprovou o processo de adaptação. 

 Referente à medida da confiabilidade da FCR-VB, 52 facilitadores de simulação realizaram o autopreenchimento do instrumento. Desses participantes, 78,85% (41) eram do sexo feminino, 73,1% (38) eram enfermeiros, 17,3% (9) eram médicos, 5,77% (3) eram farmacêuticos, 1,93% (1) eram nutricionistas e 1,93% (1) eram biólogos. Cerca de 38,5% (20) desses profissionais tinham como nível maior de formação Doutorado, 25% (13) Especialização, 23% (12) Mestrado e 13,5% (7) Pós Doutorado. Além disso, os profissionais tinham 17,19 anos de média de tempo de formação (desvio-padrão=8,24), com 10,98 anos de média de tempo de atuação no ensino (desvio-padrão=6,61), e utilizavam a simulação em sua prática de ensino/trabalho há 6,4 anos em média (desvio-padrão=5,28). A Tabela 2 apresenta a classificação dos facilitadores em simulação e os resultados das medidas de confiabilidade da versão brasileira da FCR, verificados a partir dos dados do alfa de Cronbach, mensurados para cada domínio e para a escala geral, e os valores do Coeficiente de Correlação Intraclasse (ICC). 

**Tabela 2:** - Classificação dos participantes no teste das versões pré-final e final e confiabilidade da versão brasileira da *Facilitator Competency Rubric* . Campinas, SP, Brasil, 2023

**Domínios da escala**	**Classificação**	**Teste da versão pré-final (n=33)**	**Teste de Confiabilidade (n=52)**	**Alfa de Cronbach (n=52)**	**ICC** ^*^ **(n=15)**
**Preparo**		Média ^†^ =24,54 (DP ^‡^ =7,30)	Média ^†^ =25,11 (DP ^‡^ =6,25)	0,911	0,95
	Iniciante a Iniciante Avançado	12,1% (4)	3,8% (2)		
	Competente	51,5% (17)	57,7% (30)		
	Proficiente a Especialista	36,4% (12)	38,5% (20)		
Prebriefing		Média ^†^ =14,69 (DP ^‡^ =3,99)	Média ^†^ =13,82 (DP ^‡^ =4,26)	0,876	0,96
	Iniciante a Iniciante Avançado	9,1% (3)	9,6% (5)		
	Competente	42,4% (14)	55,8% (29)		
	Proficiente a Especialista	48,5% (16)	34,6% (18)		
**Facilitação**		Média ^†^ =22,42 (DP ^‡^ =5,89)	Média ^†^ =22,82 (DP ^‡^ =4,93)	0,906	0,95
	Iniciante a Iniciante Avançado	12,1% (4)	1,9% (1)		
	Competente	27,3% (9)	53,8% (28)		
	Proficiente a Especialista	60,6% (20)	44,3% (23)		
Debriefing		Média ^†^ =30,51 (DP ^‡^ =7,45)	Média ^†^ =29,56 (DP ^‡^ =7,08)	0,947	0,97
	Iniciante a Iniciante Avançado	6,1% (2)	5,8% (3)		
	Competente	39,4% (13)	55,7% (29)		
	Proficiente a Especialista	54,5% (18)	38,5% (20)		
**Avaliação**		Média ^†^ =14,06 (DP ^‡^ =3,85)	Média ^†^ =13,76 (DP ^‡^ =3,57)	0,878	0,97
	Iniciante a Iniciante Avançado	9,1% (3)	9,6% (5)		
	Competente	48,5% (16)	61,5% (32)		
	Proficiente a Especialista	42,4% (14)	28,9% (15)		
				Total=0,980	-

* ICC = Coeficiente de Correlação Intraclasse;

† Média da pontuação por domínio;

‡ DP = Desvio-padrão por domínio

No teste da estabilidade da escala, 35 desses participantes aceitaram contribuir com o estudo, no entanto, apenas 15 retornaram com o instrumento preenchido após 15 dias de autoaplicação.

## Discussão

 A versão brasileira da *Facilitator Competency Rubric* e seu processo de adaptação obtiveram êxito em diferentes âmbitos, além de apresentar medidas confiáveis de consistência interna e validade de conteúdo. 

 A adoção de um referencial teórico e metodológico para o processo de adaptação cultural de um instrumento está diretamente associada à qualidade do resultado obtido posteriormente a todas as etapas realizadas. O rigor e a complexidade exigidos para tornar equivalentes os itens entre as versões original e adaptada de um instrumento são fundamentais para subsidiar o uso qualificado desse material. A garantia da equivalência entre as duas versões começa pela escolha de uma metodologia apropriada ^(^ ^7^ ^,^ ^15^ ^)^ . Todo esse processo foi seguido na adaptação cultural da FCR para o contexto brasileiro neste estudo. 

 Estudos que compararam métodos de adaptação cultural e suas validações evidenciaram que a etapa de *back translation* não é obrigatória, pois não há diferença significativa no resultado final do instrumento. No entanto, essa etapa pode ser útil como ferramenta de comunicação com o autor do instrumento original ^(^ ^16^ ^-^ ^17^ ^)^ . Além disso, a participação efetiva do comitê de especialistas é fundamental para a garantia das equivalências, e o retorno constante dos apontamentos gerados pela etapa do teste da versão pré-final a essa equipe multidisciplinar permite a precisão do conteúdo dos itens no comparativo entre as versões original e traduzida do instrumento ^(^ ^16^ ^-^ ^17^ ^)^ . Neste estudo, houve o cumprimento dessas duas etapas, conforme previsto no referencial metodológico escolhido ^(^ ^7^ ^)^ . 

 Ressalta-se que, para a composição do comitê de especialistas, foram buscados perfis de participantes que se adequassem ao que é recomendado pela literatura ^(^ ^7^ ^-^ ^8^ ^)^ . Um grupo multidisciplinar com atuação em diferentes frentes na simulação e em estudos de validação reforçam a qualidade e o cuidado que o presente estudo teve em garantir uma melhor validação do conteúdo do instrumento. Ademais, a escolha do valor de IVC de 0,9 incrementa a segurança e rigor do processo, como visto em outros estudos ^(^ ^18^ ^-^ ^20^ ^)^ . 

 A autora do instrumento original apresentou em seu estudo que a FCR foi projetada para ser usada na observação do facilitador de simulação, o que torna o corpo docente mais relutante em ser avaliado enquanto exerce seu papel de professor. No entanto, os resultados evidenciam que o uso autoaplicado do instrumento fornece mais credibilidade aos achados e orienta o desenvolvimento do corpo docente ^(^ ^5^ ^)^ . Portanto, o presente estudo escolheu a estratégia de auto aplicação do instrumento para obtenção dos resultados. 

 Outra importante escolha no processo de validação da FCR-VB foi a inclusão de participantes com experiência em simulação clínica e de um texto que orienta o seu preenchimento. A escolha por incluir somente pessoas com experiência no uso da simulação baseou-se no estudo alemão de tradução do instrumento, em que os autores destacaram que a inclusão de participantes com baixa ou nenhuma formação em simulação pode ter interferido nos resultados alcançados ^(^ ^21^ ^)^ . Tal observação revela que o indivíduo que preenche o instrumento necessita ter o mínimo de conhecimento sobre simulação clínica e sobre os domínios que o questionário se propõe a avaliar. 

Além disso, a inclusão do texto de orientação não interferiu diretamente no conteúdo do instrumento original, e foi sugerida também pelos participantes da pesquisa. Sem dúvida, a disponibilização de um roteiro de orientação é fundamental para assessorar o profissional que aplica a estratégia de ensino na compreensão dos objetivos para se autoavaliar como facilitador.

 Os profissionais que participaram da etapa V do estudo apresentaram sugestões pertinentes no que tange a realidade da temática. Isso reforça o que a literatura compartilha como importante: a inclusão do público alvo na coleta de dados e suas contribuições para aperfeiçoar a compreensão do instrumento, e facilitar a adesão ao seu uso dentro da prática e a obtenção de informações e resultados que sejam verídicos e de qualidade ^(^ ^18^ ^-^ ^19^ ^)^ . 

 No presente estudo, apesar de não existirem relatos anteriores sobre o tempo gasto para preencher a FCR dentro ou fora do cenário de simulação ^(^ ^5^ ^,^ ^21^ ^)^ , houve uma grande disparidade no registro de tempo para o preenchimento do instrumento. Foi verificada uma variação de dois a 40 minutos de tempo para preenchimento da escala, e emanaram comentários realizados pelos facilitadores sobre a grande quantidade de itens (29) presentes no instrumento, o que pode estar associado à maior quantidade de tempo para finalizar a autoavaliação. 

 Quanto à análise da consistência interna, os resultados do Brasil são semelhantes aos da versão alemã ^(^ ^21^ ^)^ , com valores acima de 0,8 para todos os domínios. Não foi possível comparar com os valores encontrados no estudo original, uma vez que a medida da consistência interna foi realizada de modo diferente, empregando o Coeficiente G, que mostrou resultados de bom a excelente ^(^ ^5^ ^)^ , verificados também na versão brasileira. 

 Para a medida da estabilidade da escala, verificada por meio do teste-reteste, as três versões apresentaram ICC acima dos valores de referência de 0,70 ^(^ ^14^ ^-^ ^15^ ^)^ , o que ressalta como são estáveis as medidas que o instrumento avalia em momentos distintos. 

 Este estudo não ficou isento de limitações. Na etapa do teste da versão pré-final não foi possível calcular a taxa de resposta, pois foi adotado o recrutamento em bola de neve para autopreenchimento do instrumento, o que pode ser considerado um viés amostral. Ainda, o número de indivíduos que participaram da etapa de teste-reteste para a medida da confiabilidade foi inferior ao recomendado em literatura, que prevê um mínimo de 50 indivíduos ^(^ ^8^ ^,^ ^13^ ^-^ ^14^ ^)^ . Recomendamos que, em estudos futuros e com maior tamanho amostral, sejam realizadas avaliações da estrutura da escala, por meio da análise fatorial exploratória, seguida da análise fatorial confirmatória, para confirmar a disposição dos itens nos domínios do instrumento. 

O fornecimento da versão brasileira da FCR com as propriedades de medidas testadas e validadas para o cenário acadêmico e institucional contribui para o avanço das boas práticas de simulação clínica em escopos de prática cada vez mais utilizados na área da saúde. A partir da utilização da rubrica, o facilitador poderá reconhecer seus pontos fortes e suas limitações para aplicar a metodologia da simulação, ou seja, a versão brasileira da FCR pode ser utilizada no desenvolvimento de profissionais como facilitadores em simulação clínica, ao pontuar as competências que ainda devem ser desenvolvidas. Com isso, os profissionais que pretendem atuar no papel de facilitadores de forma efetiva e eficiente poderão buscar uma capacitação mais pertinente aos seus objetivos para favorecer melhores condições de ensino e aprendizagem, utilizando todas as potencialidades que a simulação clínica oferece. Também não podemos excluir as vantagens do avanço da ciência no campo da pesquisa em simulação com a veiculação desse instrumento.

## Conclusão

O processo de tradução e adaptação da FCR seguiu criteriosamente os passos descritos como referência na literatura internacional. A inclusão desse instrumento no cenário nacional da simulação clínica brasileira pode incrementar o conhecimento sobre as competências de facilitadores e o desenvolvimento de novas habilidades para profissionais que utilizam essa estratégia de ensino. A versão brasileira da FCR mostrou-se adequada para o uso e autoavaliação. Estudos com um maior tamanho amostral e com avaliação de diferentes propriedades de medida são recomendados para aumentar a precisão e a validade do instrumento.
